# Order induces toughness in anisotropic colloidal crystal composites

**DOI:** 10.1073/pnas.2422532122

**Published:** 2025-06-11

**Authors:** Victoria Vilchez, Shitong Zhou, Florian Bouville

**Affiliations:** ^a^Department of Materials, Centre for Advanced Structural Ceramics, Imperial College London, London SW7 2AZ, United Kingdom

**Keywords:** colloidal crystal, fracture toughness, particulate composites, bioinspiration

## Abstract

Spatial ordering of matter elicits exotic properties sometimes absent from a material’s constituents. A few highly mineralized natural materials achieve high toughness through delocalized damage, whereas synthetic particulate composites must trade toughness for mineral content. We test whether ordering the mineral phase in particulate composites through the formation of macroscopic colloidal crystals can trigger the same damage resistance found in natural materials. Our macroscopic silica rod-based anisotropic colloidal crystal composites are processed fully at room temperature and pressure, reach volume fractions of mineral higher than 80%, and aided by a ductile interface, unveil toughness up to two orders of magnitude higher than bulk silica through the collective movement of rods and damage delocalization over millimeter. These composites demonstrate key design rules to break free from conventionally accepted structural materials’ properties trade-off.

## Significance

Particulate composites are central to multiple applications, from reinforced polymers in health care, electrodes in batteries, to advanced cutting tools and aerospace parts. These composites are designed today with a commonly accepted trade-off: The higher the content in stiff and brittle mineral reinforcement, the lower the composite damage tolerance and toughness. Guided by highly mineralized natural material design, we found that by making sure the mineral part presents an ordering and a high dimensional regularity, we can eliminate the presence of easy fracture paths and achieve up to 10-fold increase in toughness compared to composites with lower volume fractions and no order. This design principle opens an avenue to make high-performance composites.

Spatially ordering a material microstructure can make properties absent from its composition emerge, giving rise to the concept of metamaterials. These unusual behaviors stem from a spatial variation of properties leading to structural color (1), negative refractive index (2), or acoustic bandgaps (3). Whereas working on the effect of structure and order (4–9) in porous lattice-based materials led to numerous breakthroughs in mechanical properties, the same cannot be said about dense materials. First, it is problematic to fabricate dense and ordered microstructures at the macroscopic scale and it is difficult to predict how their mechanical properties would be affected. Toughness, a material’s resistance to fracture, is a property that depends on both the microstructure and the constituent properties (10). It is linked to a material capacity to plastically deform and its long-term durability and fatigue resistance. More importantly, a high toughness is responsible for users’ security in safety critical applications, such as aeronautics or nuclear reactors (11, 12), but also dictates the performance of functional materials necessary for the energy transition (13). Numerous microstructural changes have been tested to improve materials’ toughness and deformability by relying on mechanisms acting at the atomic (14, 15) or molecular length scale (16). Introducing order in the microstructure could provide a universal and potent way of spreading damage and delaying failure by erasing the presence of a weak path for fracture, breaking us free from the trade-off observed in structural materials (17).

Examples of such intricate control of the microstructure can be seen in natural materials and have fascinated researchers because of their combination of properties absent in man-made materials made from mainly brittle constituents (18). In nacre’s brick-and-mortar structure, toughness amplification is due to the regularity in brick-and-mortar dimensions combined with the strain hardening of the interface (19–22). Simulation work has even proved that altering even slightly this ordering of the microstructure fatally removes the damage tolerance (23). The dactyl club of the mantis shrimp (24, 25) or tooth’s enamel (26) are also associated with high hardness, damage, and wear resistance through mineral rods and organic mortar arrangement. The common denominator in all these structures is the ability of natural materials to control the local packing and to order an anisotropic mineral phase and a ductile phase over large distances (27). Numerous materials got inspired by these feats, leading to composites with impressive properties and microstructures featuring reinforcements in a wide range of sizes, from the 100 µm down to the 100 nm scale (18, 28–31). However, none of the materials made so far present an ordering of the microstructure’s constituents as good as what has been found in natural materials and thus none presented the extent of damage delocalization and toughness amplification seen in natural materials. The only examples that managed to mimic this ordering were done with macroscopic reinforcing elements, proving again that order is key to observe damage delocalization (32, 33). More generally, it is a long-standing trade-off observed in any synthetic composites: the higher the volume fraction of reinforcement in a ductile matrix, the lower the plastic deformation (34, 35). Particulates can trigger crack deflection, crack bridging, and pull out mechanisms to toughen the matrix they are embedded in. These toughening mechanisms manifest in the toughness measured as an added component to the toughness of the original matrix (36, 37). In contrast, process zone toughening delocalizes the damage over a larger volume than a single crack and thus multiplies the toughening mechanisms initially present, potentially leading to higher toughness and larger strains at failure than what can be obtained by bridging and pull-out (38). Our hypothesis, based on modeling results (23) and natural materials’ behavior (20), is that process zone toughening can only be obtained when a particulate composite’s microstructure presents a sufficient degree of order to avoid any weak path that would favor the formation of a single crack.

The only synthetic composites that come close or even exceed the order found in natural materials at the nanometers to the hundreds of microns scale are colloidal crystals. These crystals made from monodisperse particles of size ranging from a few nanometers to a micron can self-assemble into larger structures (39), forming both useful functional materials and models to study atomic structures (40). While most colloidal crystals are made of spheres, recent breakthrough in sol–gel synthesis led to monodisperse rods of silica in the micron range (41). These rods can assemble into structures similar to liquid crystals, their molecular analogue, and are already opening new ways to look at phase behavior and crystal defects in anisotropic crystals (42, 43). As most toughening mechanisms in composites rely on anisotropic elements, having anisotropic mineral particles that can assemble into colloidal crystals finally opens up a whole new field of study on the effect of order on damage resistance. There is no intrinsic limit to the volume they could reach even if the number of defects will increase with the crystal size (44). However, today most colloidal crystals are limited to a few hundreds of microns in size due to the difficulty in controlling their entropy-driven assembly and growth (44–51).

The objective of this study is thus twofold: to fabricate centimeter-sized colloidal crystals with anisotropic particles so they can be mechanically tested and to study the effect of ordering on the damage delocalization in composites. We developed a templated growth of the colloidal crystal using off-the-shelf optical parts to make millimeter-sized crystals first. The fabrication is deliberately performed fully at room temperature to explore less energy intensive ways to make composites than the ones used so far. The order and packing fraction of reinforcement in the composites is then characterized using microscopy and small angle X-ray scattering (SAXS). Finally, the anisotropic colloidal crystal composites (a-C^3^) grown to centimeter size are tested using macroscopic mechanical testing to observe and study the damage delocalization over the millimeter scale brought by the ordering and interface tailoring.

## Results

**Fig. 1.**
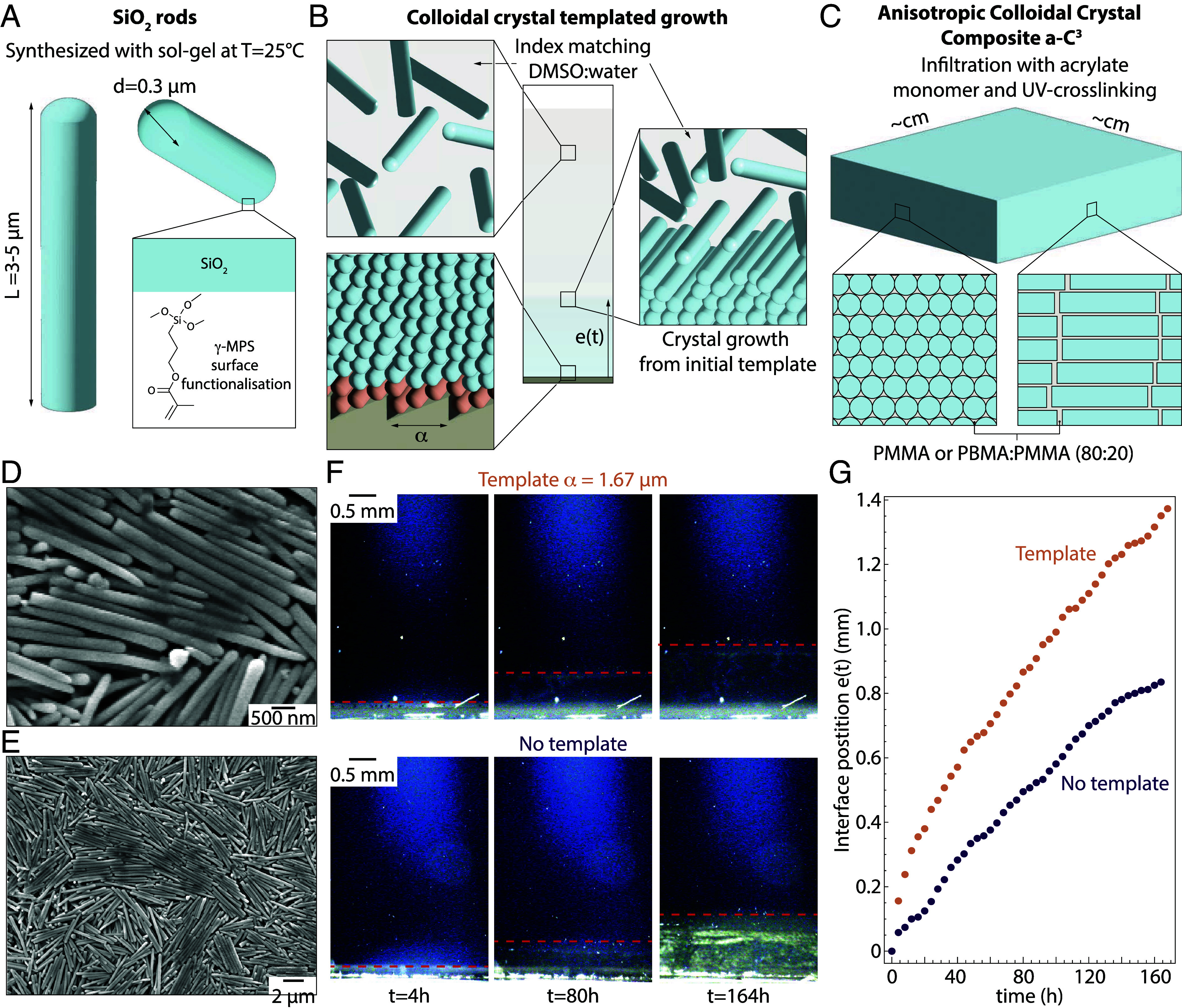
Fabrication of bulk ordered colloidal crystals from anisotropic building blocks. Schematic representation of the composite fabrication: (*A*) Sol–gel synthesis of rods and functionalization with *γ*-MPS, (*B*) templated entropy-driven assembly of the rods in DMSO-water mixture into cm-sized colloidal crystals, (*C*) infiltration with acrylate monomer after solvent-removal and cross-linking to form the a-C^3^. (*D* and *E*) SEM images of the as-synthesized rods. (*F*) Optical tracking of the crystal growth with and without template through a cross-polarized microscopy setup. (*G*) Position of the disorder-to-crystal interface as a function of time.

Fig. 1 describes the process of templating the entropy-driven self-assembly of monodisperse rods to obtain centimeter-sized a-C^3^ at close to room temperature.

Starting from the room temperature sol–gel synthesis of silica rods developed by Kuijk et al. (41), we developed a templated method to grow large-scale colloidal crystals (Fig. 1 *A*–*C*). The rods, 3 µm in length and 300 nm in diameter (*SI Appendix*, Fig. S1), are first functionalized using 3-(Trimethoxysilyl)propyl methacrylate (*γ*-MPS) to facilitate the future infiltration of acrylate monomers and strengthen the interface between the rods and the polymer interface (Fig. 1*A*). This functionalization of the surface is also what proved to be critical to avoid excessive cracking during the drying of the assembly and curing of the polymer later (*SI Appendix*, Fig. S2). Acrylate monomers can shrink by almost 10% without constrains, which can lead to the presence of residual stresses in the assembly. The rods’ dimension can be tuned by changing the reagent amounts (41) and we chose these initial dimensions to ensure a high aspect ratio while making the assembly easier, as higher aspect ratios would need a higher dilution to ensure the assembly would still start from the template and not in the suspension. A template is introduced at the bottom of the mold during the crystallization process to guide the orientation of the crystals growing and obtain a textured polycrystal of large dimension (52). The template is based on an off-the-shelf optical grating that is imprinted on a silicone substrate using a soft-lithography method. The template consists of periodic wedges angled at 33° from the horizontal and separated by 1.67 µm (*SI Appendix*, Fig. S3). In the rest of the text, the template direction refers to the direction of the grooves on the template. The rods are mixed in an index matching solvent made from dimethyl sulfoxide (DMSO) and water to decrease the magnitude of attractive interparticle forces, enabling crystallization during sedimentation. The index matching allows to decrease the van der Waals attraction forces (53), helping the formation of colloidal crystals (54, 55). A rod slotting in the wedge will lose 0.28 k_B_T of gravitational energy, where k_B_ is the Boltzmann constant and T the temperature, providing a driving force to control the orientation of the rods in the crystals growing from the template (Fig. 1*B*). The two conditions to ensure that the crystallization of the rods starts from the template and can grow from it are the following: to have an initial volume fraction of rods well below the isotropic-to-nematic phase transition and that the magnitude of the driving force for crystallization is comparable with the rod’s Brownian diffusion. The Péclet number in this condition is $P_{e}=1.95$ (*Materials and Methods*), confirming a similar contribution between advection from gravity and Brownian motion in the movement of the rods. The final step of the fabrication consists in drying the crystal formed before infiltrating it with low viscosity acrylate monomers. The composites are then polymerized under UV light, forming large-scale a-C^3^.

We first confirmed the formation and size of the rods from the sol–gel synthesis and their capacity to self-assemble upon sedimenting using SEM (Fig. 1 *D* and *E*). Having the rods dispersed in an index matching solvent allows to have a fully transparent solution when the rods are in the isotropic phase, which becomes translucent as the rod concentration increases. We used an optical setup with cross polarizers to follow the growth of the crystal over time with or without the template (Fig. 1*F*). The crystal growth front is visible in both configurations, however the sample without template presents a visibly more heterogeneous structure, with multiple grains of different colors present after 164 h. This suggests that multiple crystals with different orientations are present in the nontemplated conditions, each crystal changing the light polarization differently. SEM images of nontemplated assembly showed that these crystals reach dimensions of a few hundreds of microns (*SI Appendix*, Fig. S4*A*). The position of the interface as a function of time provides quantitative information on the crystal growth. The templated growth presents a faster initial growth compared with the nontemplated one, with a crystal thickness three times larger after 20 h. However, the growth speed appears similar after this initial burst. The faster initial growth in presence of the template points toward a faster nucleation of the crystal with the template, an effect also seen in other colloidal crystal systems and thought to occur in seeded crystal growth of any kind (56).

Finally, we demonstrate that crystals of several millimeters in height can be obtained from a templated growth after several days and that their optical properties suggest a uniform orientation of the rods throughout the whole sample. This process is scalable to larger sample sizes by enlarging the template area and longer sedimentation time. While this growth time can seem long, we manage to obtain composites in the mm^3^ range in two weeks, from rod synthesis to composite fabrication. It seems a necessary price to pay to obtain the high degree order in the microstructure and to keep the embedded-energy necessary to fabricate these a-C^3^ as low as possible.

We can a priori grow cm-scale colloidal crystals from this simple process at close to room temperature, but the local structure and orientational control over macroscopic distance is key to study the mechanical properties.

We confirmed using small-angle X-ray scattering (SAXS) and electron microscopy that we can control the orientation of rods over the millimeters scale and thus grow some of the largest textured colloidal polycrystals made with a local packing close to the theoretical limit.

**Fig. 2.**
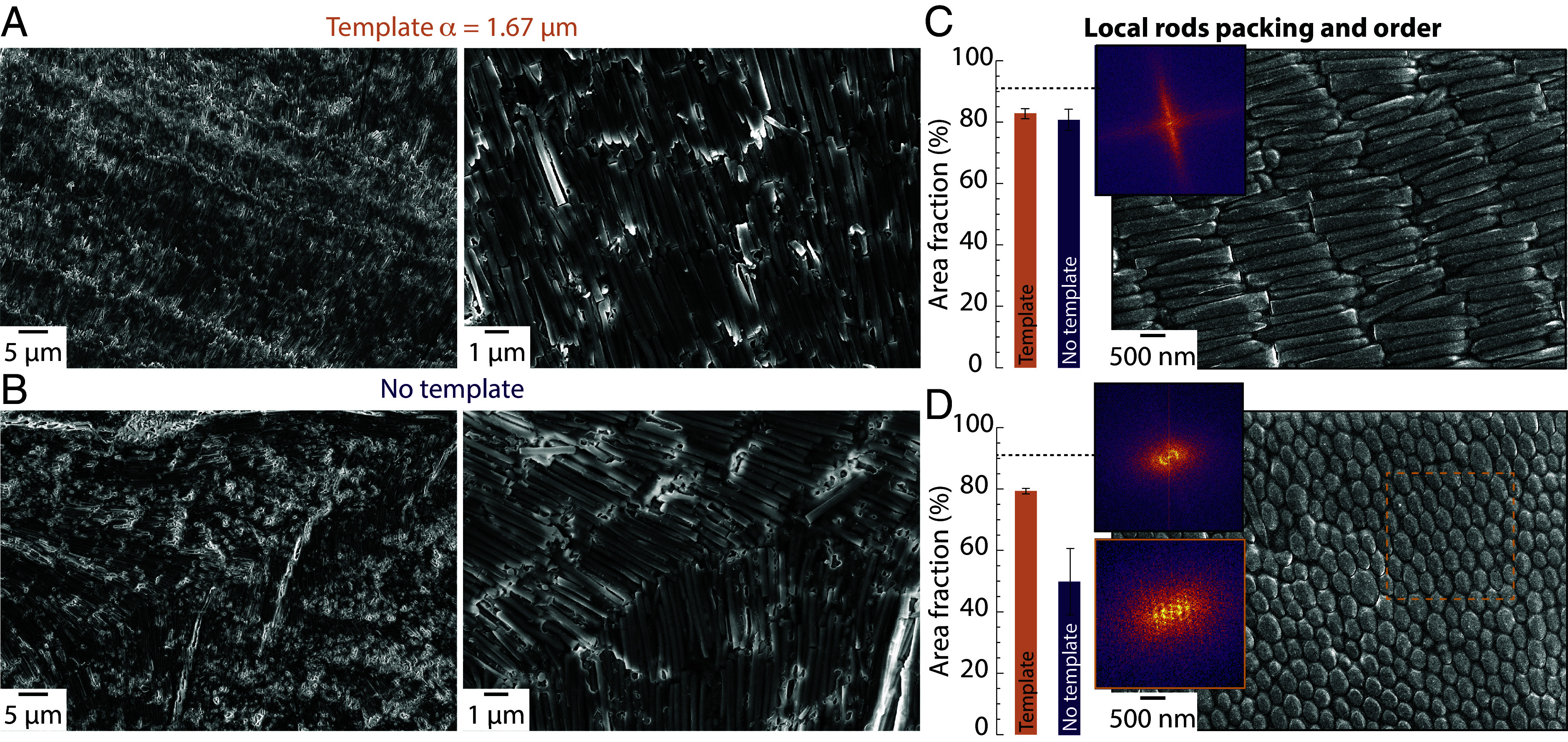
Short-range order of the rods in a-C^3^. SEM images of the structure of composites fabricated (*A*) with a template and (*B*) without. SEM images of ion-polished cross-section of the a-C^3^ grown from a template with cross-section taken (*C*) in the wedge direction and (*D*) perpendicular to the wedge direction. *Insets* are FFT of the images or of the highlighted area in the image. The dotted line at 91% in the histograms is the theoretical maximum volume fraction of rods.

SEM images of the top surface of composites grown with and without template show that the rods are indeed self-assembled into a smectic phase in the composite, with layers of rods stacked into columns with their ends almost aligned. While the sample grown with a template shows an alignment of rods over tens to hundreds of microns (Fig. 2*A* and *SI Appendix*, Fig. S4*B*), the sample grown without a template displays changes in orientation of the rods over distances around tens of microns. Multiple topological defects expected from free growth of anisotropic colloidal crystals are present in the nontemplated sample, with for instance a disclination line visible in Fig. 2*B*. Even in the templated sample the rods’ main orientation changes over long distances; however, the orientation changes around a common direction. This observation is coherent with what we expect from the templated growth, with multiple crystals growing at the same time from the template with a common rod orientation.

Using ion-polishing, the smectic packing of the rods becomes even more apparent, while the polymer layer with a thickness of around 30 nm can also be more easily seen (Fig. 2*C*). A fast Fourier transform (FFT) of the image displays a cross-shaped feature, revealing that the stacking of rods in the image displays a preferred orientation, but also that the ends of the rods form an additional pattern oriented at 90° from the rod stacking direction. An SEM image of the ion-polished cross-section taken perpendicular to the template direction provides further insight into the packing of the rods (Fig. 2*D*). The FFT of the whole image reveals a periodicity in the distance between the rods, with a ring visible along a defined direction. Rods can even form a more ordered structure locally, with an FFT highlighting the presence of local hcp packing. The packing of rods was quantified using image analysis of multiple images and reached 82% ± 2% and 64% ± 15% for the templated and nontemplated composites (images for nontemplated samples are in *SI Appendix*, Fig. S5) respectively. The templated crystal packing values are close to the theoretical limit for hard rods of 91 vol% [estimated based on the hexagonal packing of perfect cylinder (57)], which is higher than most composites fabricated around room temperature where the packing is limited by reinforcement polydispersity, and as high as some composites made using multiple pressing and heating steps (31, 58).

**Fig. 3.**
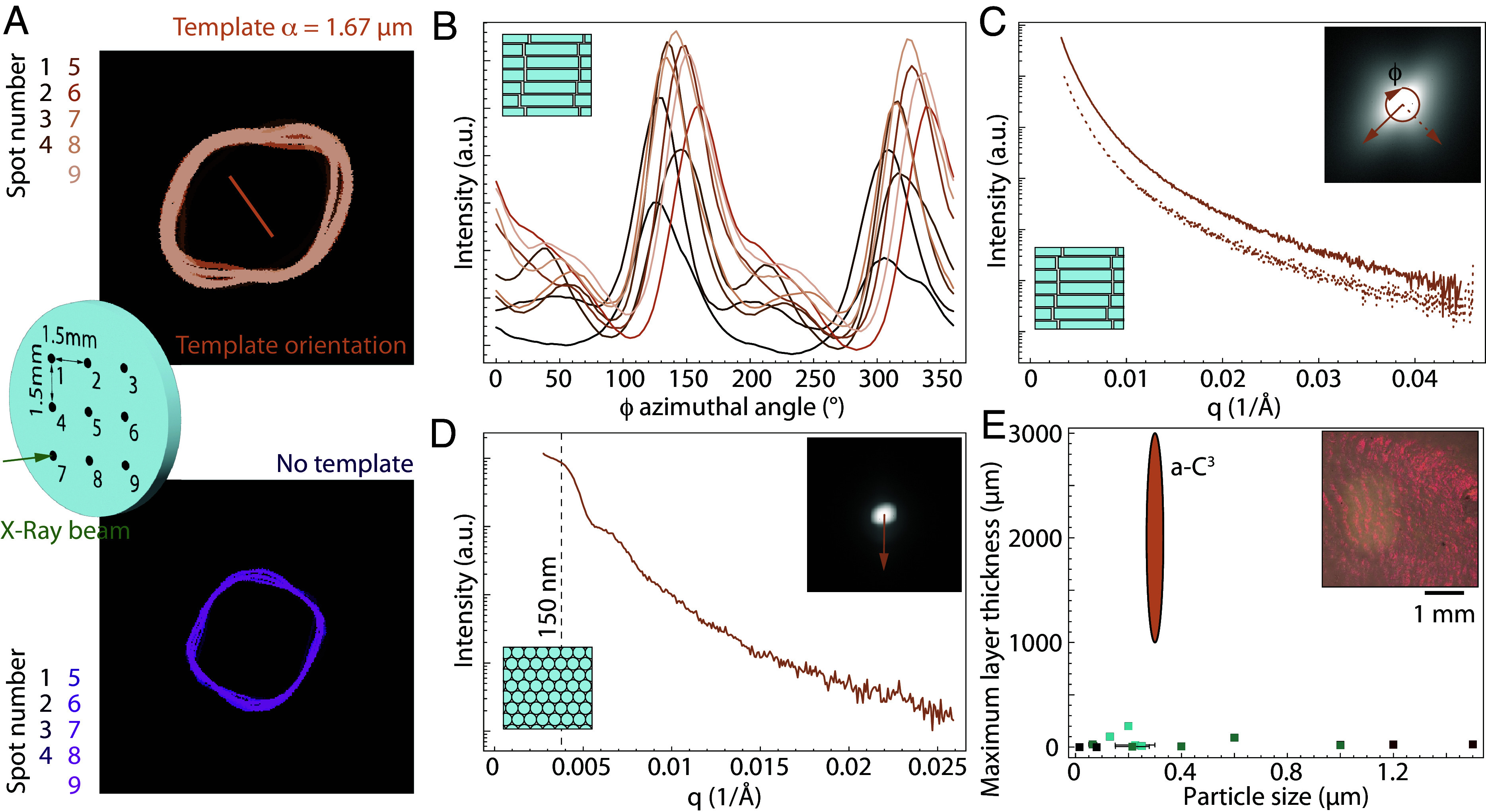
Long-range order of the rods in a-C^3^. (*A*) Line representing the intensity at half maximum of the SAXS signal made at nine different spots on sample grown with and without template with the X-rays traversing the sample in the direction of the crystal growth. The line in the top image represents the orientation of the wedge of the template. (*B*) Integrated intensity as a function of the azimuthal angle ϕ for all scans taken on the sample grown with a template. (*C*) Intensity as a function of wavenumber in the direction of the template and perpendicular to it. (*D*) Intensity as a function of wavenumber with the X-ray going along the direction of the template. (*E*) Comparison of the size of the crystal grown with the silica rods in this work with other methods to grow large-size colloidal crystals with spheres. (*Inset*) optical microscope image of an a-C^3^ taken in the direction of the sample growth before infiltration. Data from literature color coded based on the process used: templated growth in brown (44, 45, 50), directional evaporation/shear in green (47, 48, 51, 59, 60), sedimentation in blue (46, 61–63), and triggered self-assembly in solvent in black (49, 64).

While SEM provides evidence of both the short- and long-range control over the rod packing and orientation provided by the templated growth method, obtaining samples with this ordered structure over millimeter to centimeter size is necessary to measure the effect of order on the mechanical properties of a-C^3^. We decided to use SAXS to characterize the orientation and order over larger volumes. The SAXS beam probes a disk of 200 µm in diameter through the whole sample thickness, and we recorded the SAXS pattern at nine different spots spaced by 1.5 mm, covering a total zone of 3 × 3 mm^2^ (Fig. 3*A*). The isolines at 50% of scattered intensity at the nine positions for a sample grown with and without template with the X-rays taken along the growth direction are represented in Fig. 3*A*. All the patterns obtained in the templated sample present a preferred orientation at 90° from the direction of the template, whereas the sample without template displays a more isotropic response. The SAXS patterns are reminiscent of the FFT obtained in Fig. 2*C* and *SI Appendix*, Fig. S6, with a cross shape visible although less clearly, indicating that the rods’ columns are less ordered in the bulk than locally but confirming the presence of a smectic phase in the whole sample. The azimuthal profiles confirm the common orientation (Fig. 3*B*), with a major peak visible around ϕ = 150° in all patterns. The position of this primary peak across the nine scans indicates an alignment within ±8° of the rods’ orientation around the template direction. This dispersion around a common orientation is confirmed using image analysis on the sample surface (*SI Appendix*, Fig. S7). These results prove that our a-C^3^ composites present a common orientation over several millimeters, i.e., over scales four orders of magnitude higher than the average rod length. The radial profiles along the primary direction, at 90° from the template direction, and the secondary direction, at 0° from the template direction, do not show any features (Fig. 3*C*), proving that there is no periodicity of the rods’ columnar arrangement over long distances. However, the cross-sectional radial profiles taken when the X-rays traverse the sample in the direction of the template orientation show rings at a wave vector of 0.0042 Å^−1^ (Fig. 3*D*) along a specific direction. This wave vector corresponds to around 150 nm in real space. We attribute this ring to the second-order diffraction generated by the periodicity in rod-to-rod distance corresponding to their 300 nm diameter. This ring appears only in specific direction in the SAXS pattern (Fig. 3 *D*, *Inset*) as in the results from electron microscopy (Fig. 2*C*), confirming that the long-range order in the rod stacking is anisotropic. These three SAXS curves can further be fitted using a cylinder form factor, confirming the dimensions of the rods measured as well as their alignment and polydispersity (*SI Appendix*, Fig. S8 and Table S1).

The study of the microstructure of the a-C^3^ proves that the templating method successfully orients the rods over areas of several millimeters squared and thicknesses of several millimeters. These bulk anisotropic textured colloidal polycrystals are some of the largest reported, with thicknesses one order of magnitude higher than other templating methods used with isotropic colloids so far (Fig. 3*E*). The testimony of this control of the rod orientation over macroscopic distances can also be seen in their optical properties, with a visible shimmering (*SI Appendix*, Fig. S9) and even the presence of structural colors when observed under a microscope (Fig. 3 *E*, *Inset*) when no polymer is present between the rods.

We can now fabricate millimeter-scale colloidal textured polycrystals, but the final mechanical properties will be dictated by the local structure and the rods and polymer properties.

**Fig. 4.**
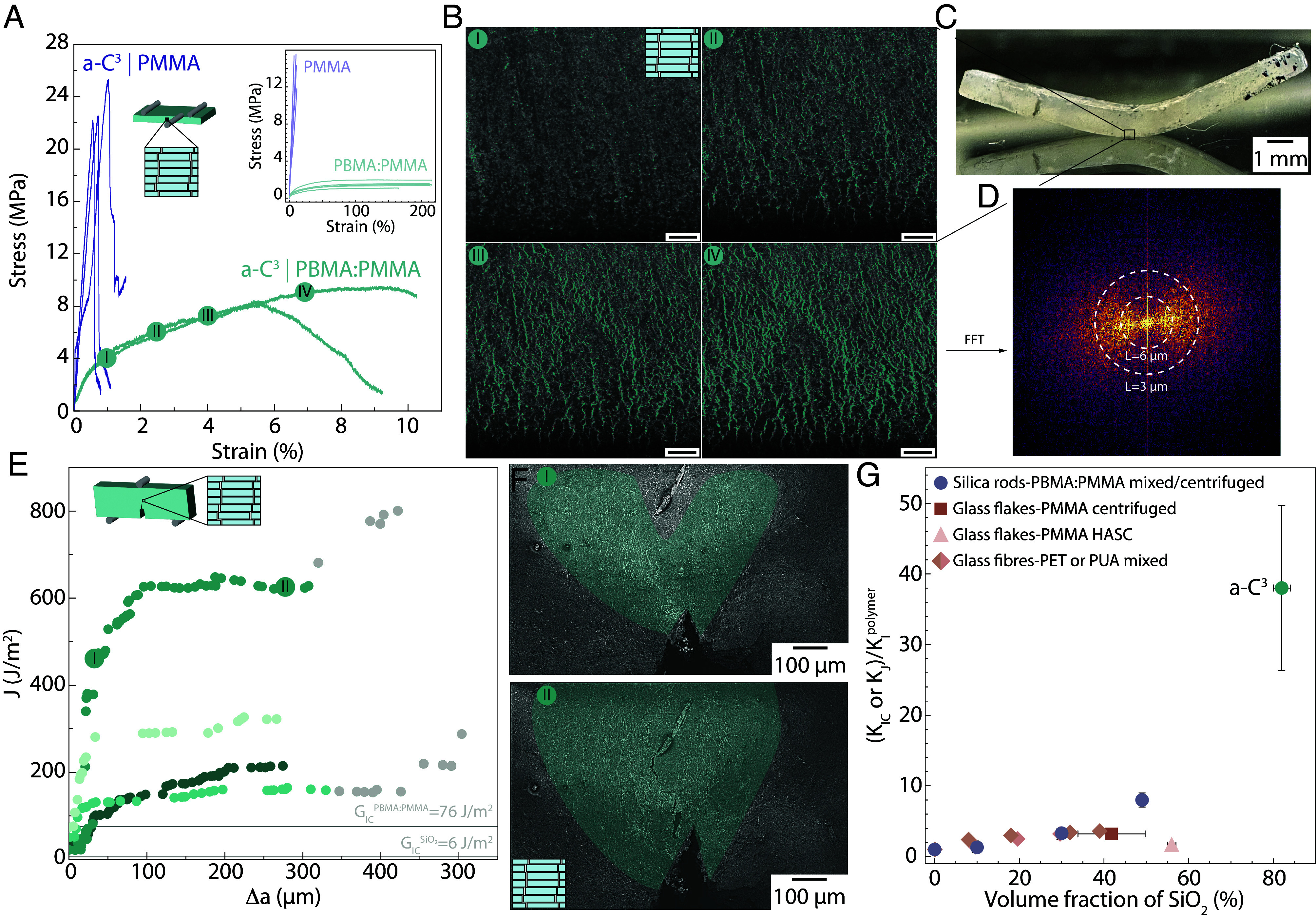
Mechanical macroscopic behavior of a-C^3^. (*A*) Stress–strain curves for a-C^3^ composites tested in bending with two polymer interfaces. (*Inset*) Stress–strain curves of the pure polymers used as interfaces in the composites tested in tension. (*B*) SEM images of the zone in tension taken at different strains during in situ bending test of a-C^3^|PBMA:PMMA. Cracks are highlighted in teal. (Scale bar 20 µm.) (*C*) Image of the a-C^3^|PBMA:PMMA after bending test. (*D*) FFT of the cracks network visible in the bottom part of the sample. (*E*) R-curve measured from single-edge notch bending tests of a-C^3^|PBMA:PMMA. Grayed symbols represent values beyond the ASTM-recommended crack extension limit. Each teal hue represents a sample tested. Gray lines represent the toughness of PBMA:PMMA and of silica. (*F*) SEM of the crack front taken at different crack lengths during the in situ fracture test. Microcracked area highlighted in teal. (*G*) Stress intensity factors of particulate composites $K_{I}$ or $K_{J}$ normalized with the polymer toughness $K^{\mathrm{polymer}}$ for a-C^3^|PBMA:PMMA and composites made by mixing and centrifuging rods at various volume fractions of silica rods, compared with particulate composites made in the literature from mixing glass fibers in polymer resins (68, 69), centrifugation (70), and heat assisted slip casting (71).

Now that we can obtain macroscopic highly mineralized composites with short- and long-range order of reinforcement, we are finally able to test how this microstructure responds to mechanical loads and damage. Inspired by natural materials, we know that the properties of the interface are instrumental in enabling damage tolerance. We test this hypothesis by using two different acrylate-based polymers with vastly different properties as the composite interface: PMMA, a commonly used linear elastic brittle polymer (65–67), and a mixture of PBMA:PMMA at a 80:20 ratio in mass that presents a plastic strain of 200% at the expense of rigidity and strength (Fig. 4*A*). We performed flexural tests in situ in an SEM to visualize the damage in the highly mineralized composites with a fine spatial resolution.

While the presence of PMMA at the interface leads to an almost brittle behavior of the composites (Fig. 4*A* and *SI Appendix*, Fig. S10), the introduction of a deformable interface enables plastic strain as high as 10% in this 80 vol% mineral composite. This high strain value is even more impressive if we compare it with the ones obtained in particulate composites: Regardless of the composition, size, and shape of the ceramic reinforcements, as well as strain at failure of the interface, particulate composites present strains at failure within 0 to up to 2% in the 60 to 98 vol% of reinforcement range (*SI Appendix*, Fig. S11). We attribute this 5 to 10-fold increase in strain at failure to the order introduced in the reinforcement through colloidal crystal assembly and the high strain at failure of the polymer at the interface. The large strain obtained is macroscopically visible as our samples are significantly bent after testing (Fig. 4*C*).

To confirm the role of the rods’ ordered packing in the large deformation at failure of the composites, we observed the damage on the face in tension during flexural testing using electron microscopy in situ (Movie S1). Only a few cracks are visible at the composite yield point around 1% strain (Fig. 4 *A* and *B*), but they quickly multiply once the macroscopic strain reaches 4%. The composite at 7% strain presents a dense network of cracks, with a spacing on the order of the rods’ length and apparently following the columns formed in the smectic phase. We validate this with an FFT of the crack network obtained at 7% strain, and the image confirms that the crack network is oriented vertically and presents some periodicity that falls in the range of the rod’s length and thus smectic column width (Fig. 4*D*). While the polymer phase alone presents cracks when tested in bending, the crazing lines are less numerous and spaced by distances in the 30-micron range (*SI Appendix*, Fig. S12). The network of cracks follows the smectic arrangements of the rods’ assembly, leading to a multitude of fine cracks spread out in the microstructure instead of a single crack that would concentrate the stress locally and lead to the failure of the specimen.

This ordered architecture thus seems powerful at avoiding the creation of a major crack, a capacity that can be further probed during fracture testing. The resistance to crack propagation of the a-C^3^|PBMA:PMMA was quantified using conventional macroscopic single-edge notch bending test and electron microscopy to follow the damage in real time. The toughness of the composites as function of crack extension, also called R-curves, are plotted in Fig. 4*E* along with the toughness of the bulk silica and of the pure polymer. All the composites present a rising R-curve, with the energy release rate increasing on average by a factor 10 over the first 70 µm. The rapid increase in toughness with the initial crack extension can be linked with the stability of the fracture process: the higher the toughness, the higher the necessary stress to reach an unstable fracture (72). The final toughness of the composite reached values that are 20 to 100 times higher than bulk silica and 1.9 to 8 times higher than the pure matrix, with energy release rates reaching 120 to 600 J/m^2^ compared to 6 J/m^2^ for bulk silica (73, 74) and 76 J/m^2^ for the PBMA:PMMA matrix (*SI Appendix*, Fig. S13). We further confirmed using larger samples that these toughness values are not sample-size dependent (*SI Appendix*, Fig. S14). Crack deflection, twisting, or bridging and pull-out toughening mechanisms could not lead to such a high toughness amplification. Crack deflection can only lead to a factor 2 in toughness increase and this only for a maximum deflection angle of 90° (see formula and demonstration in the Supplementary discussion and information). Regarding crack bridging and pull-out, we use a micromechanical model developed for fiber bridging in short fiber composites (75), later adapted to high reinforcement volume fraction natural brick-and-mortar composites (20). The model is built on the assumption that to open a crack, one in two rods will need to be pulled-out. This is the assumption leading to the highest toughening from bridging as it corresponds to a nematic phase for the rod assembly, a perfect smectic phase producing no bridging and pull-out. Following this assumption, it is possible to express the closure stress from bridging and thus the additional component to the stress intensity factor that needs to be applied to open the crack as a function of the properties of the matrix being sheared during pull-out. We obtained (see supplementary discussion for the details) an added stress intensity factor from bridging $K_{b}$ expressed as $K_{b}=\sqrt{E_{\mathrm{comp}}\cdot \frac{1}{4}\cdot \frac{L_{\mathrm{rods}}}{d_{\mathrm{rods}}}\cdot \tau_{i}\cdot u_{m}}$, with $E_{\mathrm{comp}}$ the composite Young’s modulus, $L_{\mathrm{rods}}$ the average length of the rods, $d_{\mathrm{rods}}$ the average diameter of the rods, $\tau_{i}$ the shear strength of the matrix, and $u_{m}$ the distance at which the sliding becomes zero. Applying this bridging model leads to an additional toughness of $K_{b}=0.05\;MPa.m^{\frac{1}{2}}$, representing a 2.6 times increase in stress intensity factor compared with the matrix. However, this cannot explain the value of toughness the a-C^3^|PBMA:PMMA reaches, with a toughness in stress intensity factors 10 to 20 times higher compared with the toughness that could be obtained by bridging only.

The toughness increase is directly linked with the formation of a damage zone in front of the crack full of rod columns sliding (Fig. 4*F* and Movie S2). This damage zone increases in size in front of the crack and prevents any further propagation of the major defect, proving the capability for damage delocalization of the a-C^3^ structure. Even after the damage zone is fully developed and the main crack starts to extend, its propagation is surrounded by a process zone reaching 0.5 mm, thus making a hundred rods shift within the process zone width, each shift forming a crack (Fig. 4*F*). We can demonstrate that the toughness values reached by the a-C^3^|PBMA:PMMA composites can be explained by the size of the process zone, the stress at which the smectic phase columns are sliding and the distance over which they slide. If we assimilate the material behavior within the process zone to a perfectly elasto-plastic material which yields at a stress $\bar{\sigma }$ and presents an average plastic strain of $\bar{\epsilon }$, it is possible to express the toughness $J^{\lim}$ obtained when the process zone is fully developed and reaches a width of $w^{\lim}$ using a J-Integral approach (36) as $J^{\lim}=\;2\;\cdot \;-\bar{\sigma }\;\cdot \;-\bar{\epsilon }\;\cdot \;w^{\lim}$. Now if we assume that the smectic columns move at stress equal to $\sigma_{\mathrm{comp}}^{\mathrm{yield}}$ measured in bending (Fig. 4*A*), thus $\bar{\sigma }=\sigma_{\mathrm{comp}}^{\mathrm{yield}}$ and that the strain is obtained mainly by opening cracks of width $\Delta L_{\mathrm{crack}}$ (*SI Appendix*, Fig. S15), neglecting the elastic strain of the rods and matrix, we obtain $\bar{\epsilon }\approx \Delta L_{\mathrm{crack}}/L_{\mathrm{rods}}$. Using our in situ results, we can measure the size of the fully developed process zone $w^{\lim}\approx 300\;\mu m$ (Fig. 4*E*) and an average crack opening of $\Delta L_{\mathrm{crack}}=0.710\;\mu m$, we can estimate a theoretical toughness from process zone size of $J_{\mathrm{th}}^{\lim}=682J/m^{2}$. This value is remarkably similar to the measured toughness value (Fig. 4*E*) of $620\;J/m^{2}$ and confirms that the toughening originates from the sliding of more than 200 columns of rods within the width of the process zone.

In order to demonstrate the higher toughness increase reached by the process zone toughening compared with toughening obtained from bridging and pull-out in disordered particulates composites, we produced a series of samples in which the rods do not present any ordering and measured their fracture properties. While it is not possible to obtain a volume fraction of rods as high as the one reached in a smectic phase, we used a centrifugation procedure developed for glass flakes polymer composites (70) to reach a higher volume fraction than with mixing (*SI Appendix*, Fig. S16). We provide the stress intensity factors normalized by the critical stress intensity factor of the PBMA:PMMA matrix as a function of volume fraction of reinforcement in Fig. 4*G*. This normalization allows to compare the toughness increase coming from the structure obtained with the room temperature fabricated rods and matrix with more energy intensive to produce but higher-performance composites from the literature. The composites produced at low volume fraction of rods by mixing present a toughness up to 3.3 higher than the matrix, in line with what would be expected from bridging (cf. toughening estimation from bridging above). This toughness increase is also similar to what is achieved in short glass fibre reinforced composites with various polymer matrices (68, 69) (Fig. 4*G*). Using centrifugation increases the volume fraction of rods up to 49% [confirmed with Thermogravimetric Analysis (TGA), *SI Appendix*, Fig. S17] and the relative toughness increases up to eight times. This increase is more than two times higher than results from the literature based on centrifugation (70) and heat assisted slip casting (71). Introducing order in the rods’ assembly provides a 40-fold increase in toughness compared to the matrix alone, and almost a 10-fold increase compared to composites with disordered rods. This extent of toughness amplification is visible only in a few natural materials, mainly nacre, and is associated with the collective sliding of bricks within a millimeter-sized zone (20). Finally, we show that the intrinsic properties of these composites can be further improved by increasing the properties of the constituents. Heat treating the silica rods to remove any residual solvent at 600 °C (*SI Appendix*, Fig. S17) leads to an increase in the rods’ and a-C^3^ composites’ stiffness by almost a factor 2 (*SI Appendix*, Figs. S18 and S19). a-C^3^ made from stiffer rods present a toughness increased also by almost a factor 2 (*SI Appendix*, Fig. S20), demonstrating that this concept can be extended to stiffer and stronger materials and thus to intrinsic higher structural properties. In addition, as we do not observe rod failure in our samples, their length could be increased, leading to higher stress transfer and potentially stronger composites (76).

## Conclusion

In conclusion, we introduced order into particulate anisotropic composites using a combination of principles from colloidal crystal, traditional crystal growth, liquid crystal, and composite fabrication. Because of that, our a-C^3^ are manufactured entirely at close to room temperature and pressure, reach macroscopic sizes, and still present up to 80 vol% of mineral reinforcements. There are no physical limits to the crystal size that can be obtained through this technique, the growth speed would limit the part to centimeters in thickness, whereas the lateral dimensions are only limited by the template and could reach larger dimension than the centimeter. The monodispersity of the rods and the entropy-driven templated assembly lead to a smectic microstructure that is present at short range but also at long range as confirmed by a combination of microscopy and diffraction results. The rods’ highly ordered structure triggers toughening mechanisms at local and macroscopic length scales only seen in natural materials so far. The composites present a toughness 20 to 100 times higher than silica (and 1.9 to 8 times higher than the matrix) and a macroscopic strain at failure of up to 10%, breaking free of the expected trade-off between high reinforcement content and high deformability and toughness in particulate composites. We established that this capacity to delocalize damage over large volumes is directly rooted in the order of the microstructure and the polymeric interface deformability. The materials produced here are intrinsically interesting to unveil further the influence of microstructure periodicity on mechanical properties at the macroscopic scale. But more importantly, the effect we provide here can be applied to any composition and for any application where high toughness and high mineral content is crucial, from solid electrolyte energy storage devices to high-performance structural materials for extreme environments, such as nuclear fusion reactors or space shuttle protection systems.

## Materials and Methods

### Materials.

*γ*-MPS (98%), ammonia solution (28% in water, >99.99%), 2,2-dimethoxy-2-phenylacetophenone (DMPA, 99%), DMSO, methyl methacrylate, (MMA, 99%), 1-pentanol (ACS reagent, >99%), polyvinylpyrrolidone (PVP, Mw = 40,000 g/mol), sodium citrate dihydrate (>99%) and tetraethyl orthosilicate (TEOS, 98%) were purchased from Aldrich-Merck. Ethanol (>99.5% Ph. Eur., USP) was purchased from VWR. Polydimethylsiloxane (PDMS, SYLGARD™ 184 silicone elastomer kit) was purchased from Dow. n-butyl methacrylate (n-BMA, 99%) was purchased from Thermal Scientific.

### Synthesis of Colloidal Silica Rods.

The synthesis is a sol–gel process reported by Kuijk et al. (41). 12 g of PVP was dissolved in 120 mL of 1-pentanol by stirring for several hours in a 500 mL glass bottle. Then, 12 mL 1-pentanol, 12 mL ethanol, 3.36 mL deionized water and 0.80 mL of a solution of 0.18 M sodium citrate in deionized water were added to the bottle. The bottle was shaken by hand to form the emulsion of water droplets in 1-pentanol stabilized by PVP and sodium citrate. 2.70 mL of ammonia solution was added and the bottle shaken again. Afterward, 1.20 mL of TEOS was added and the bottle was shaken one last time, before being left still to react overnight. Once all the TEOS had reacted, the synthesized rods underwent a series of centrifuging (5804, Eppendorf) and washing steps under the following cycle: The mixture was first centrifuged at 1,500 g for 45 min before the supernatant was poured out. It was then redispersed in ethanol to be centrifuged at the same speed for 15 min two times. This step was repeated two more times in water and another time in ethanol. The rods were redispersed in ethanol with three final centrifuging steps at 700 g for 15 min. To remove the excess solvent trapped in rods during sol–gel synthesis, dried rods were annealed at 600 °C for 1 h.

### Rods Functionalization.

Once the supernatant in the last centrifuging step was removed, the centrifuge tube was placed in an oven at 60 °C for at least 15 min to fully evaporate the ethanol. The dry mass of rods was weighed and redispersed with a weight fraction of 10 wt% in a solution of γ-MPS:ethanol 1:2 in volume. The solution was stirred at room temperature for 24 h, then centrifuged and washed twice in ethanol with runs at 1,500 g for 15 min. Finally, the rods were dried at 60 °C for 15 min.

### Assembly into Colloidal Crystals.

The dry rods were weighed and resuspended in an index matching solution. Index matching was obtained by dispersing rods in DMSO (n = 1.479 at 20 °C) then adding deionized water (n = 1.333 at 20 °C) dropwise until the sol became transparent. The volume fraction of rods in solvent was chosen to be 2 vol% to ensure the rods are in the isotropic phase before sedimenting. A volume ratio of 10:2.1 DMSO:H_2_O had to be used during sedimentation to match the refractive index of functionalized rods, yielding an effective refractive index of n = 1.45. Once the rods had been dispersed in DMSO and H_2_O, the system was left to self-assemble and sediment for at least 7 d. During the assembly of rod-like colloids, the competition between sedimentation under gravitational forces and Brownian diffusion can be first estimated by the gravitational Péclet number (77):

$$Pe=\frac{4\pi \Delta \rho gR^{4}}{3k_{B}T},$$

for spherical particles of radius *R*, with Δρ the difference in density between the solvent and particles, *g* the acceleration of gravity, $k_{B}$ the Boltzmann constant and *T* the temperature. By assimilating rods to spheres of radius equal to their radius of gyration $R=\sqrt{\frac{d_{\mathrm{rods}}^{2}}{2}+\frac{l_{\mathrm{rods}}^{2}}{12}}\;=\;752\;\mathrm{nm}$ (78) with Δρ=ρrods-ρDMSO = 1,710 − 1,100 = 610 kgm3 and T = 293 K, *P_e_* is 1.95. The density of the rods was measured using a pycnometer (50 mL, Blaubrand).

In order to improve the assembly of crystals in a single orientation on a larger scale, unidirectional templating was used. The templates were made of PDMS by duplicating the pattern on an optical grating with groves spaced by 1.67 µm (ruled diffraction gratings with 600/mm grating, Thorlabs). These PDMS-imprinted stubs were then placed at the bottom of polypropylene syringes (5 mm diameter and 75 mm height, 1 mL Plastipak, BD) for producing crystals in 5 mm diameter disks during sedimentation. The piston and conical tip of the syringe were removed before the stubs were sealed with the syringe using epoxy glue (Araldite Instant 90 s). For upscaling the size of the crystals, glass capillaries (14 × 7 × 70 mm and 24 × 8 × 100 mm, CM Scientific) were used as the containers for sedimentation. To prevent the crystals from sticking to the wall of the glass capillaries after resin infiltration, fluorinated ethylene propylene film (FEP Release Film Liner, Elegoo) was put on the walls of the glass capillaries.

### Composite Manufacturing.

After 7 d of sedimentation, a sediment of 2 to 3 mm height had formed at the bottom of the cylinder. The supernatant was removed and the remaining solvent evaporated by placing the sample in a vacuum oven (OV-11, Jeio Tech). To evaporate the water first, the oven was kept at ambient pressure and heated at 70 °C for 4 h. Then, vacuum was pulled down to −0.1 MPa while keeping the temperature at 70 °C to evaporate the DMSO. Once the sediment was dry, it was removed from the oven and infiltrated with the monomer and photoinitiator mix. Two acrylate monomers were tested, MMA and BMA. The photoinitiator was DMPA. For infiltration with BMA and/or MMA, the monomers were mixed with 10 wt% DMPA. The monomer mix was then slowly added dropwise from the top side of the sediment, leaving enough time for capillary forces to draw the liquid toward the bottom of the sediment without trapping air bubbles. The infiltration of a liquid with a refractive index close to the one of silica rods led to a change of translucency of the sediment upon impregnation, allowing for a visual confirmation of uniform infiltration of the resin. The infiltrated composite was then consolidated by curing the acrylate resin in a UV chamber (Asiga Flash, Asiga) at 365 nm for 30 min. For comparison, composites with 10 vol% and 30 vol% silica rods were prepared by mixing the monomer and functionalized rods. The mixture was cast in a mold and cured under the same condition as a-C^3^|PBMA:PMMA. A higher volume fraction of rods was introduced in the polymer by centrifuging a mixture containing 2 vol% silica rods (the same concentration as for self-assembly) at 2,000 g for 20 min, following the centrifugation procedure for glass composites (70). The supernatant was removed before the centrifuged composites were cured for 30 min in the UV chamber.

### Characterization of Crystal Growth Using Optical Microscopy under Polarized Light.

Polarized light microscopy was used to monitor the sedimentation of silica rods in the DMSO/H_2_O solvent and observe the nucleation of crystalline domains. Imaging the sample under polarized microscopy allows to observe 2 mechanisms in 1 setup. First, the observation of birefringence, characterized by the apparition of two refractive indices in a material depending on the light propagation direction. Birefringence can change the polarization of light, meaning that light can still pass through a crossed polarizer-analyzer. In the case of the silica rod composites, birefringence could occur from the optical anisotropy of aligned rods in the nematic and smectic phases (79). In addition to birefringence, Bragg diffraction can occur due to the periodical slits formed by rods as they assemble into nematic or smectic phases. In the case of silica rods of spacing approximately equal to their diameter d = 300 nm, the colloidal crystal diffracts in the visible light range. Because Bragg diffraction can also modify the polarization of light if the latter is not contained in the plane of diffraction, it results that diffracted light can pass through a crossed polarizer-analyzer too.

To set up the polarized microscopy experiment, glass capillaries of 50 mm in length with a cross-section of 0.5 mm × 5 mm and a wall thickness of 0.350 mm were used for rod sedimentation (VitroTubes, VitroCom). The glass capillary was bonded to the PDMS template using a plasma bonding technique. To ensure maximum surface adhesion, the bottom of capillaries was first polished down to 5 µm using SiC paper (CarbiMet S, Buehler). The PDMS stub and polished capillary were placed in the chamber of a plasma cleaner (Femto basic unit type D, Diener Electronic, Germany). An oxygen plasma was generated at a gas flow of 15 sccm and a power of 50 W and held for 30 s. The two parts were bonded straight after being taken out of the chamber. The capillary was then filled with the solution of rods at 2 vol% in DMSO and H_2_O mixture and placed between a polarized light source (BL-ZW1, Dino-Lite, UK) and an optical microscope with a built-in polarizer (AM7013MZT, Dino-Lite, UK). The polarizer of the light source was oriented at 90° to the polarizer of the microscope in order to achieve complete extinction. Images were captured every 4 h during a total time of 7 d.

### Characterization of Microstructure Using Scanning Electron Microscopy.

The microstructure of the silica rod composites was first characterized using SEM. Before imaging, the composites were embedded in epoxy (EpoThin 2, Buehler) and polished down to 1 µm using diamond suspensions (DiaPro, Struers). To further flatten the surface and image only the contrast between the silica rods and the acrylate resin, some samples were polished using broad argon ion beam milling (PECS II, Gatan) for 30 min at 4 keV and 6°. A conductive coating of 10 nm of chromium was sputter coated on the samples prior to imaging (Q150T S, Quorum). Samples were imaged in the SEM at acceleration voltages of 5 kV for the secondary electron detector and 10 kV for the backscattered electron detector (Auriga CrossBeam, Zeiss).

### SAXS.

Since sending X-rays on a colloidal crystal of lattice spacing of hundreds of nm results in diffraction at very small angles, the silica rod composites were characterized using SAXS at the Diamond Light Source synchrotron facility using the DL-SAXS beamline. Silica rod composites of 5 mm diameter and a few millimeters in height were prepared and cut in slices of 1 mm thickness, with the rods either in-plane or in the cross-sectional view. In addition, a blank sample made of pure acrylate resin was used as a control to ensure that the observed signal was solely due to the interaction of silica rods with the incident beam. Slices were placed in a 6 mm disc solid rack. Scans were taken in ambient conditions, using an Excillium Ga MetalJet source of 9.2 keV and a EIGER2 R 1 M detector with a pixel size of 75 µm. Each sample was imaged taking a grid of 3 × 3 measurements per sample, separated by 1.5 mm in each direction. Two sets of measurements were taken with the sample-to-detector distance set to 1 m and 4.5 m. Data processing and visualization was conducted using the DAWN software (80).

### TGA.

TGA (NETZSCH STA 449F1) was conducted by heating samples to 1,300 °C at 10 °C/min in air to measure the mass loss.

### Mechanical Testing.

The strength and toughness of the freshly prepared a-C^3^|PBMA:PMMA were determined by in situ three-point bending using a 300 N microtest stage (MT300, Deben, UK Ltd.), with a span of 11 mm and a test speed of 0.1 mm/min. The composites were tested on the day of the cross linking, we observed a small time dependency of the PBMA:PMMA mechanical properties and thus kept this timing between cross-linking and testing constant. The tensile surface and the surface to be imaged were polished down to 1 µm using diamond suspensions. Three bars of 14 × 2 × 1 mm^3^ for each composition (rods infiltrated with PMMA and PMMA:PBMA = 20:80) were tested for strength and four bars of 14 × 3 × 1 mm^3^ for toughness. To assess the size effect of the composites, three larger samples (24 × 4 × 1 mm^3^) were tested with a span of 21 mm. One of the bar for PMMA:PBMA = 20:80 was tested with an unloading step after 3% strain to demonstrate that there was plastic strain and is plotted separately in *SI Appendix*, Fig. S21. The stage was placed in a Zeiss Sigma FEG-SEM to monitor crack propagation. The crack propagation of pure PBMA:PMMA resin was observed for comparison purpose. The cracks in the SEM images during the test were segmented and reconstructed using interactive top-hat in Avizo 9.3. For the measurement of toughness, single-edge notched beams were prenotched with a 0.25 mm diamond wafering blade, which were then sharpened manually using a razor blade to obtain a notch length $a_{0}$ comprised between 0.4 W < $a_{0}$ < 0.5 W.

To determine crack resistance curves, nonlinear elastic fracture mechanics was considered, including the contribution of elastic and plastic deformation. This was calculated according to ASTM E1820 with the *J*-integral method, where the elastic contribution $J_{\mathrm{el}}$ was given by,

[1] $$J_{\mathrm{el}}=K_{i}^{2}/E^{\prime },$$

where $K_{i}$ is the fracture toughness at a given crack length $a_{i}$ and its corresponding force $F_{i}$, $E^{\prime }$ is $E/(1-\upsilon^{2})$, *E* is the Young’s modulus and υ is Poisson’s ratio.

[2] $$K_{i}=\frac{3F_{i}L}{2BW^{\frac{3}{2}}}\times {\left(\frac{a_{i}}{W}\right)}^{\frac{1}{2}}\times \frac{1.99-\frac{a_{i}}{W}\times \left(1-\frac{a_{i}}{W}\right)\left(2.15-3.93\frac{a_{i}}{W}+2.7{\left(\frac{a_{i}}{W}\right)}^{2}\right)}{\left(1+2\frac{a_{i}}{W}\right){\left(1-\frac{a_{i}}{W}\right)}^{\frac{3}{2}}},$$

where *B* is the bar thickness, *W* is the width, *L* is the span. The plastic contribution $J_{\mathrm{pl}}$ was calculated in an iterative manner, expressed with:

[3] $$J_{pl(i)}=\left[J_{pl\left(i-1\right)}+\frac{1.9\left(A_{pl\left(i\right)}-A_{pl\left(i-1\right)}\right)}{b_{i-1}B}\right]\times \left[1-\frac{0.9\times \left(a_{i}-a_{i-1}\right)}{b_{i-1}}\right],$$

where $b_{i-1}$ is the uncracked ligament width. The $A_{\mathrm{pl}}$ was evaluated at each increment of crack length, with the plastic area given by,

[4] $$A_{pl\left(i\right)}=A_{pl\left(i-1\right)}+\left(F_{i}+F_{i-1}\right)\frac{v_{pl\left(i\right)}-v_{pl\left(i-1\right)}}{2},$$

where $v_{pl\left(i\right)}$ is the plastic part of the force–displacement given by $v_{\left(i\right)}-F_{i}C_{i}$, $v_{\left(i\right)}$ is the displacement and the compliance $C_{i}$ was determined from:

[5] $$C_{i}=\frac{1}{\mathrm{EB}}{\left(\frac{S}{W-a_{i}}\right)}^{2}\times \left[1.193-1.98\frac{a_{i}}{W}+4.478{\left(\frac{a_{i}}{W}\right)}^{2}-4.443{\left(\frac{a_{i}}{W}\right)}^{3}+1.739{\left(\frac{a_{i}}{W}\right)}^{4}\right].$$

As a comparison, the strength and toughness of the pure resin (PMMA and PMMA:PBMA) were measured on Zwick/Roell Z010 at a test speed of 1 mm/min. To determine the strength and Young’s modulus, the dogbone samples were machined using a CO_2_ laser cutter (Omtech SH-G3020 40 W) in the dimension described as Type V in ASTM D638 (81). The toughness of the resin was evaluated using a single-edge notch tension test. The stress intensity factor is given by ref. 82:

[6] $$K_{I}=\frac{F}{B\sqrt{W}}\sqrt{2\text{tan}\frac{\pi a}{2W}}∙\frac{0.752+\frac{2.02a}{W}+0.37{\left(1-\text{sin}\frac{\pi a}{2W}\right)}^{3}}{\text{cos}\frac{\pi a}{2W}},$$

where *F* is the peak applied load, *B* is the thickness of the bar, *a* is the notch length and *W* is the width of the bar.

The Young’s modulus of the composite was measured using instrumented indentation. The surfaces of the samples were polished down to 1 µm with diamond suspensions and then flattened using broad ion beam milling for 30 min at 4 keV and 6°. They were mounted in the nanoindenter (NanoTest Vantage, Micro Materials) equipped with a diamond Berkovich tip (Young's modulus *E_i_* = 1,141 GPa, Poisson’s ratio $v_{i}\;=\;0.07$, Micro Materials). Load-depth data were acquired by indenting the samples 30 µN using a loading time of 2 s and unloading time of 1 s. 225 indents per sample were performed. The acquired load-depth curves were processed using the Oliver-Pharr method (83). The stiffness *S* of the material can be deducted by fitting the upper part of the unloading curve with a power law and taking its derivative. Then the reduced Young’s modulus $E_{r}$ can be calculated and the Young’s modulus of the sample $E_{s}$ is linked to $E_{r}$ by,

[7] $$\frac{1}{E_{r}}=\frac{\left(1-{v_{s}}^{2}\right)}{E_{s}}+\frac{\left(1-{v_{i}}^{2}\right)}{E_{i}},$$

where $v_{s}$ is the Poisson’s ratio of sample (0.17 for amorphous silica). We found a Young’s modulus of the a-C^3^|PBMA:PMMA is $E_{a-C^{3}}=4.1\pm 2.0\;\mathrm{GPa}$.

## Supplementary Material

Appendix 01 (PDF)

Movie S1. *In situ* SEM flexural testing in 3 points bending configuration of an a-C^3^ | PBMA:PMMA composite

Movie S2. *In situ* SEM fracture testing in SENB configuration of an a-C^3^ | PBMA:PMMA composite

## Data Availability

All study data are included in the article and/or supporting information.
