# Supplementary material for: Order induces toughness in anisotropic colloidal crystal composites
Source: Proc Natl Acad Sci U S A. 2025 Jun 11;122(24):e2422532122. doi: 10.1073/pnas.2422532122 (PMC12184424; doi:10.1073/pnas.2422532122)
Supplement: Supplementary file 1 — Appendix 01 (PDF) [file pnas.2422532122.sapp.pdf]

Order induces toughness in anisotropic colloidal crystal composites

Victoria Vilchez[1]†, Shitong Zhou[1]†, Florian Bouville[1]*

[1]Centre for Advanced Structural Ceramics, Department of Materials, Imperial College London, London (UK)

†These authors contributed equally to this work.

*Corresponding author. Email: f.bouville@imperial.ac.uk

Supplementary movies description

Movie S1: *In situ* SEM flexural testing in 3 points bending configuration of an a-C^3 | PBMA:PMMA composite
Movie S1: *In situ* SEM fracture testing in SENB configuration of an a-C^3 | PBMA:PMMA composite

Supplementary discussions and information

<u>Estimation of the energy lost during rod slotting in the template wedge</u>

A rod of length L and diameter d slotting in a wedge of height h will lose an amount of energy $E_{slot} = V_{rod}\,(\rho_{SiO_2} - \rho_{DMSO})\,g\,h = \frac{\pi d^2}{4}\,L\,(\rho_{SiO_2} - \rho_{DMSO})\,g\,h$, with $\rho_{SiO_2} = 1710\,kg/m^3$ and $\rho_{DMSO} = 1100\,kg/m^3$ the density of silica and DMSO respectively, $g = 9.81\,m.s^{-2}$ the gravitational acceleration. Taking $L_{rods} = 2.5\,\mu m$ and $d_{rods} = 300\,nm$, we obtain that $E_{slot} \approx 0.28\,k_B T$ where k_B is the Boltzmann constant and T room temperature.

<u>Estimation of the contribution of different toughening mechanisms to the toughness of the anisotropic colloidal crystal composites a-C^3</u>

Starting from a 2D nematic microstructure as depicted in the first figure of the main manuscript, when the crack encounters a rod it can deflect at most at 90°. We can use Cotterel and Rice's (1) approach to estimate the toughness decrease for a deflected crack and thus the additional stress intensity factor needed to keep the crack from propagating.

$$K_I^{deflected} = c_{11}(\theta)K_I + c_{12}(\theta)K_{II}$$

Where $c_{11}(\theta)$ and $c_{21}(\theta)$ are coefficients that depend on the deflection angle θ. With $\theta = 90°$, the stress intensity from the deflected crack is decreased by almost a factor 2, so deflection could increase the toughness by two with respect to the fracture toughness of the interface in the composite $K_{IC}^{interface}$. We do not observe a large-scale deflection in our composite fracture with the ductile interface PBMA:PMMA, meaning that the toughness amplification by deflection is lower than 2.

Deflection alone cannot explain the toughness amplification measured in our composites. Using the work from Barthelat *et al.* (2), we estimate how much the bridging by periodically spaced rods can increase the composite toughness. This estimation of the bridging force and toughness is only valid in a 2D cross section passing by the whole diameter of the rods, as the area of rods being deformed will be smaller in other cross sections. Thus, this estimation is the upper bound of the toughness obtained through bridging. For one bridging rod of length L_{rods} and diameter d being pulled out by at maximum half of its length from the other side of the crack, we estimate the closure force to be $F = \frac{L_{rods}}{2} \cdot \tau_i$ with τ_i the shear strength of the polymer at the interface between rods. The smectic ordering would lead to no pull-out length as the rods' end would be all aligned within the whole composite. The microstructure and SAXS results show that the rods present various overlap with adjacent rod layers due to the imperfect smectic packing and non-uniformity of the rods' lengths. We use half of the length of the rods as pull-out length as an exaggeration of the actual pull-out length, so we obtain the maximum bridging toughness amplification. By the same rationale, we estimate that one in two rods are

bridging the crack, which is a strong overestimation from what we observed during *in situ* fracture measurements. The continuous bridging traction homogenised over the bridged crack can be expressed as $t(u) = \frac{F}{2d} = \frac{1}{4}\frac{L_{rods}}{d_{rods}}\ \tau_i$.

The bridging toughness amplification in terms of energy release first G_b can then be obtained using (3):

$$G_b = 2\int_0^{u_m/2} t(u)du = \frac{1}{4}\frac{L_{rods}}{d_{rods}}\tau_i\, u_m$$

Where u_m is the sliding distance at which the cohesion becomes zero. We can get a conservative estimate of the interfacial strength as $\tau_i = \sigma_i/2$, with $\sigma_i = 1MPa$ the strength of the PBMA:PMMA interface. The sliding distance u_m can be estimated from the *in situ* fracture test images to around 0.5 µm, leading to an upper estimation of the bridging toughness of $G_b = 0.625\, J/m^2$. Putting the value in terms of stress intensity factor using $K = \sqrt{G\,E}$ with G energy release rate and E the Young's modulus of the composite, we obtain $K_b = 0.05\ MPa.m^{\frac{1}{2}}$.

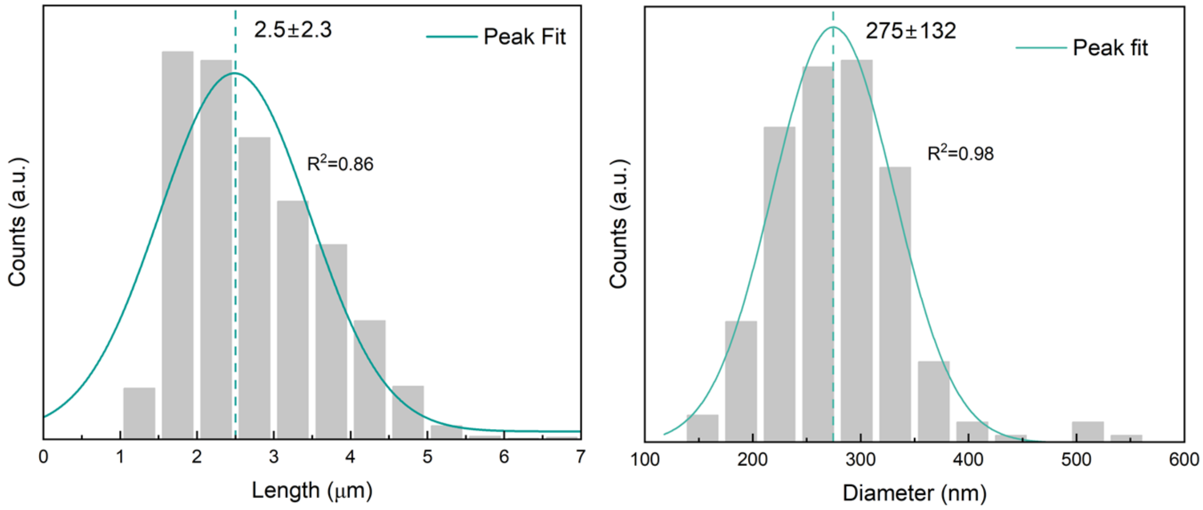

Figure S1. Distribution of rod length and diameter from sol-gel synthesis.

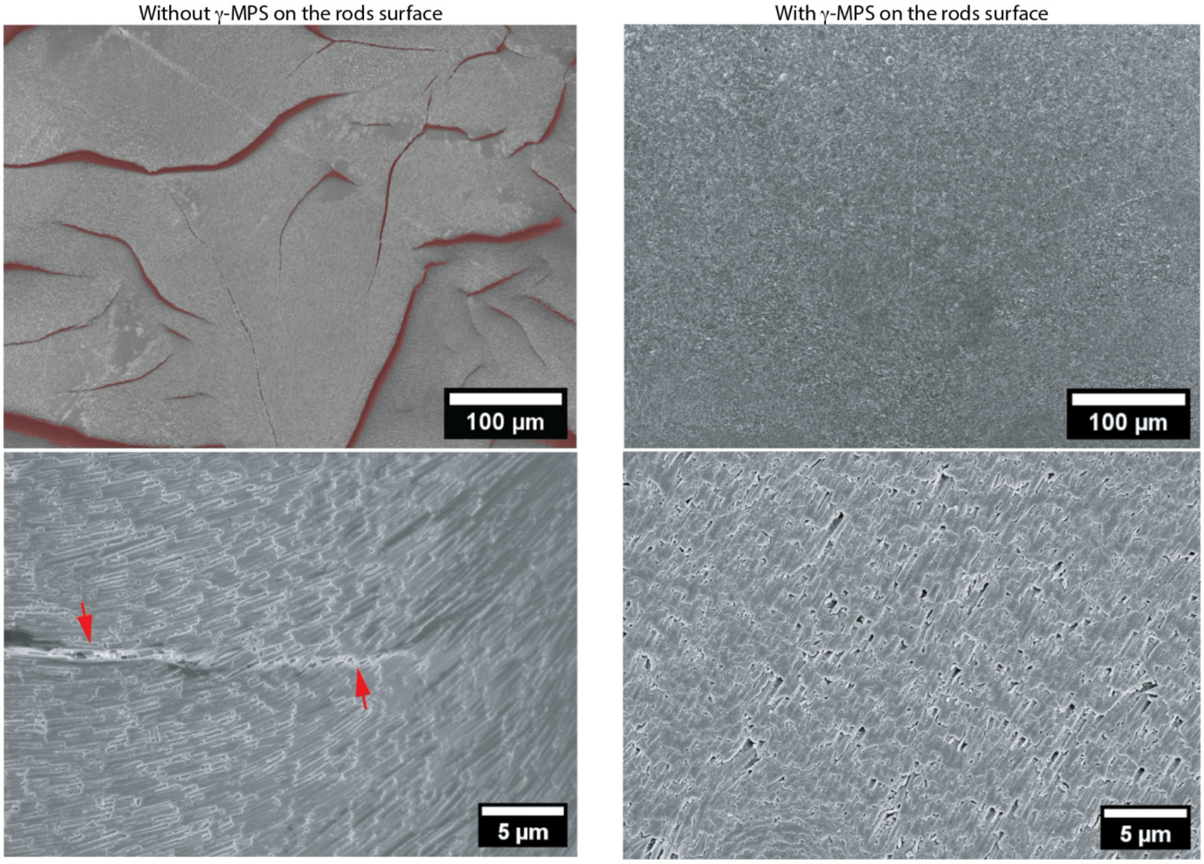

Figure S2. SEM images of self-assembled silica rods without and with γ-MPS functionalisation. Cracks are highlighted in red and red arrows on both SEM images without γ-MPS.

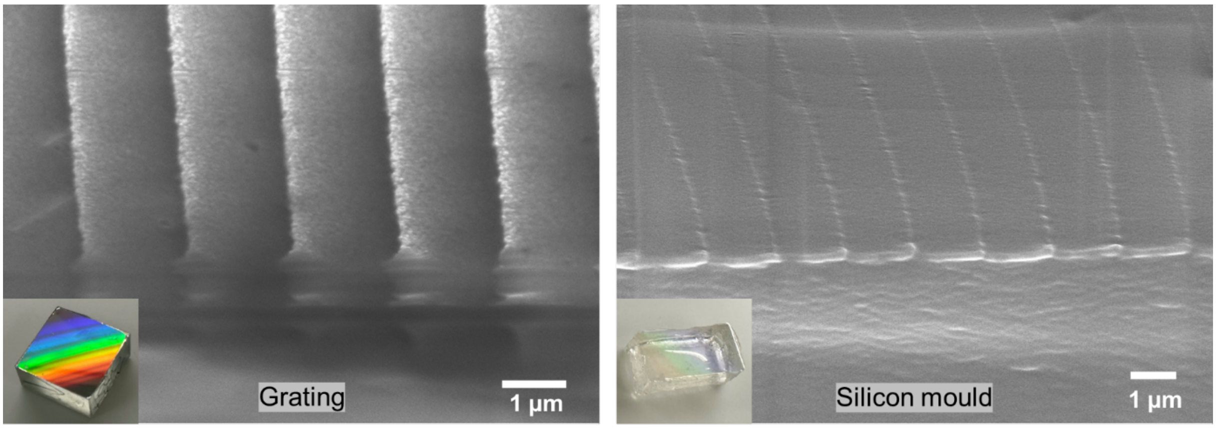

Figure S3. SEM images of the grating and the silicon mould, inserts show the iridescence from Bragg diffraction.

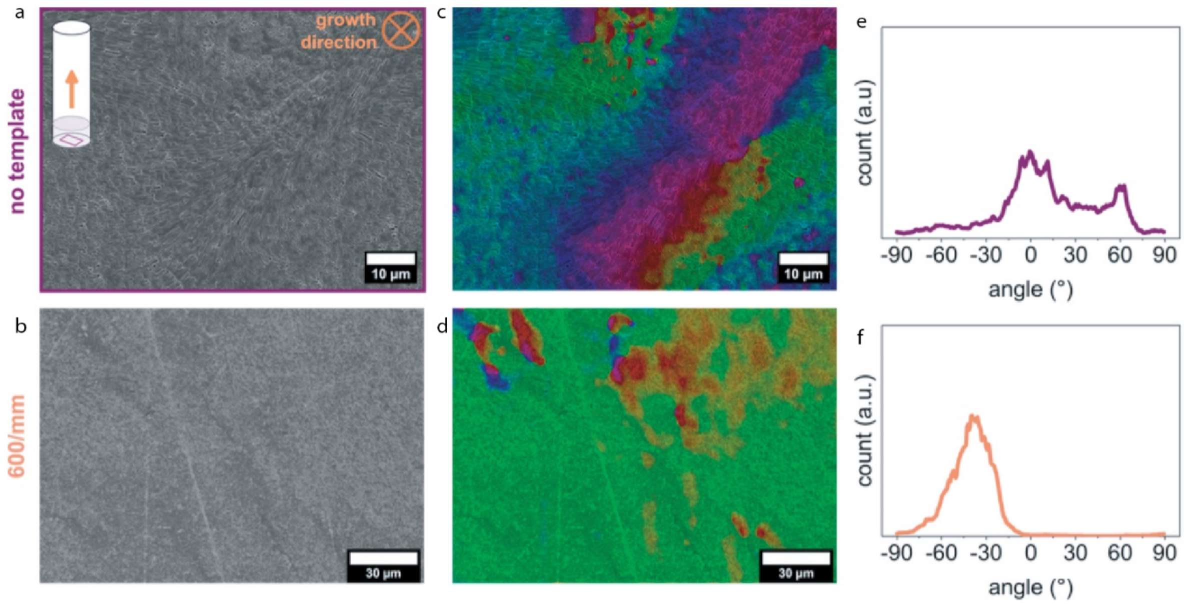

Figure S4: Characterisation of long-range order using image analysis. (a-b) SEM grayscale images of samples without template and with a 600/mm grooved template. (c-d) Corresponding colour maps obtained with the OrientationJ plugin in ImageJ showing the local rod orientation. (e-f) Corresponding angle distributions.

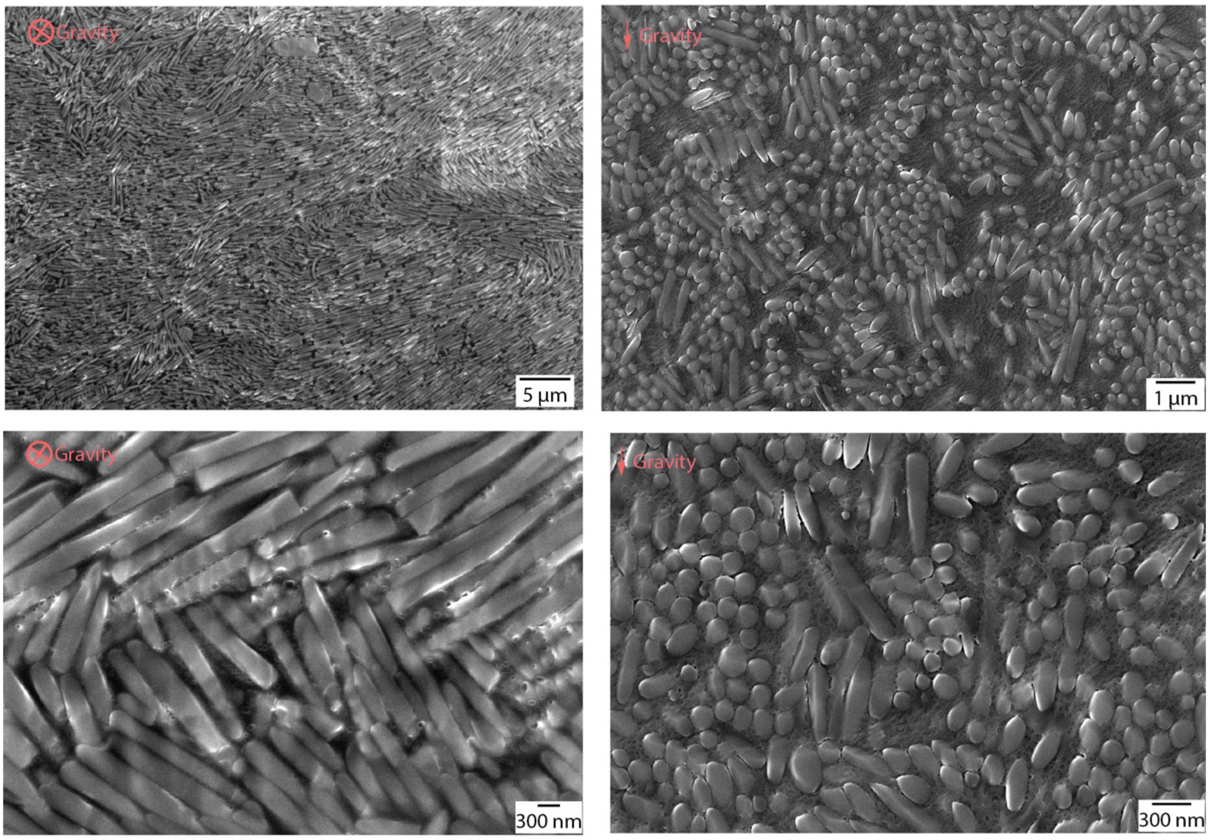

Figure S5. SEM images of ion-polished composite surfaces for non-templated samples.

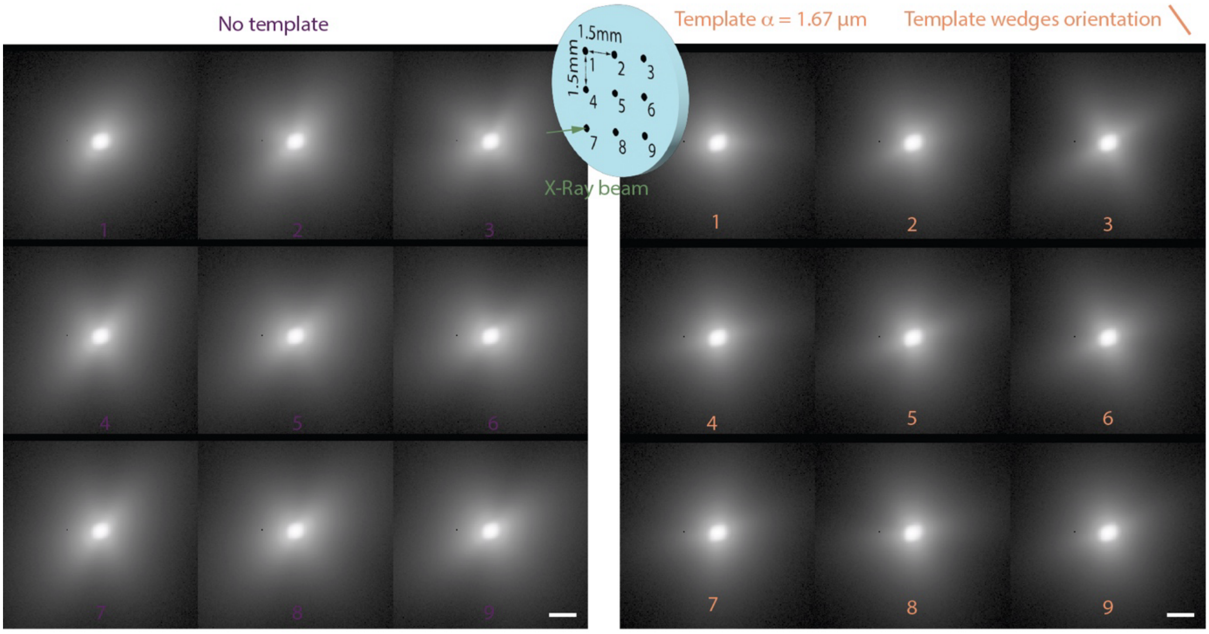

Figure S6. SAXS patterns. Scale bars represent 0.05 nm^{-1}.

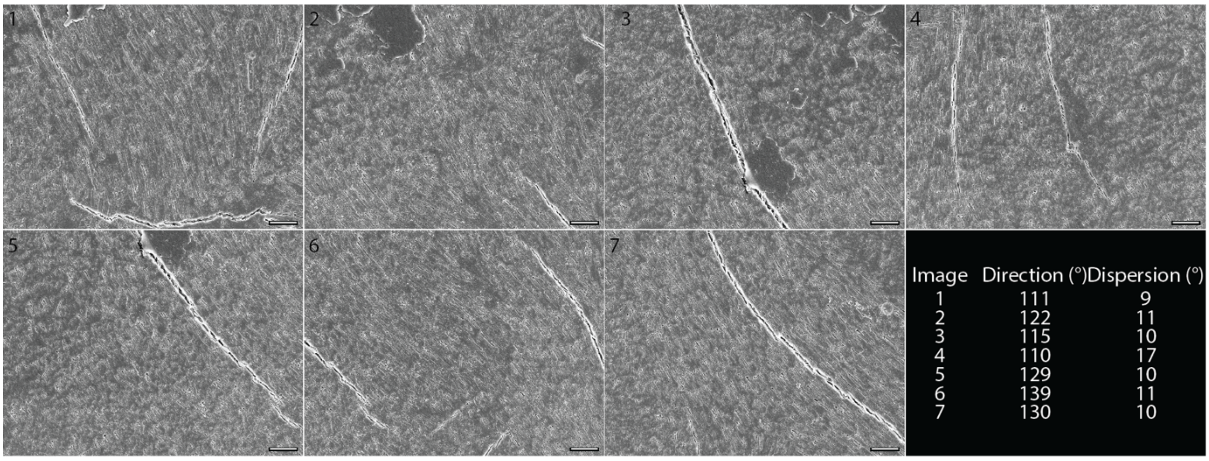

Image	Direction (°)	Dispersion (°)
1	111	9
2	122	11
3	115	10
4	110	17
5	129	10
6	139	11
7	130	10

Figure S7: SEM images of the surface of the sample tested in SAXS with a template and no γ-MPS and results of the orientation measured through image analysis (plugin Directionality from the Fiji(4)). Scale bar 5 µm.

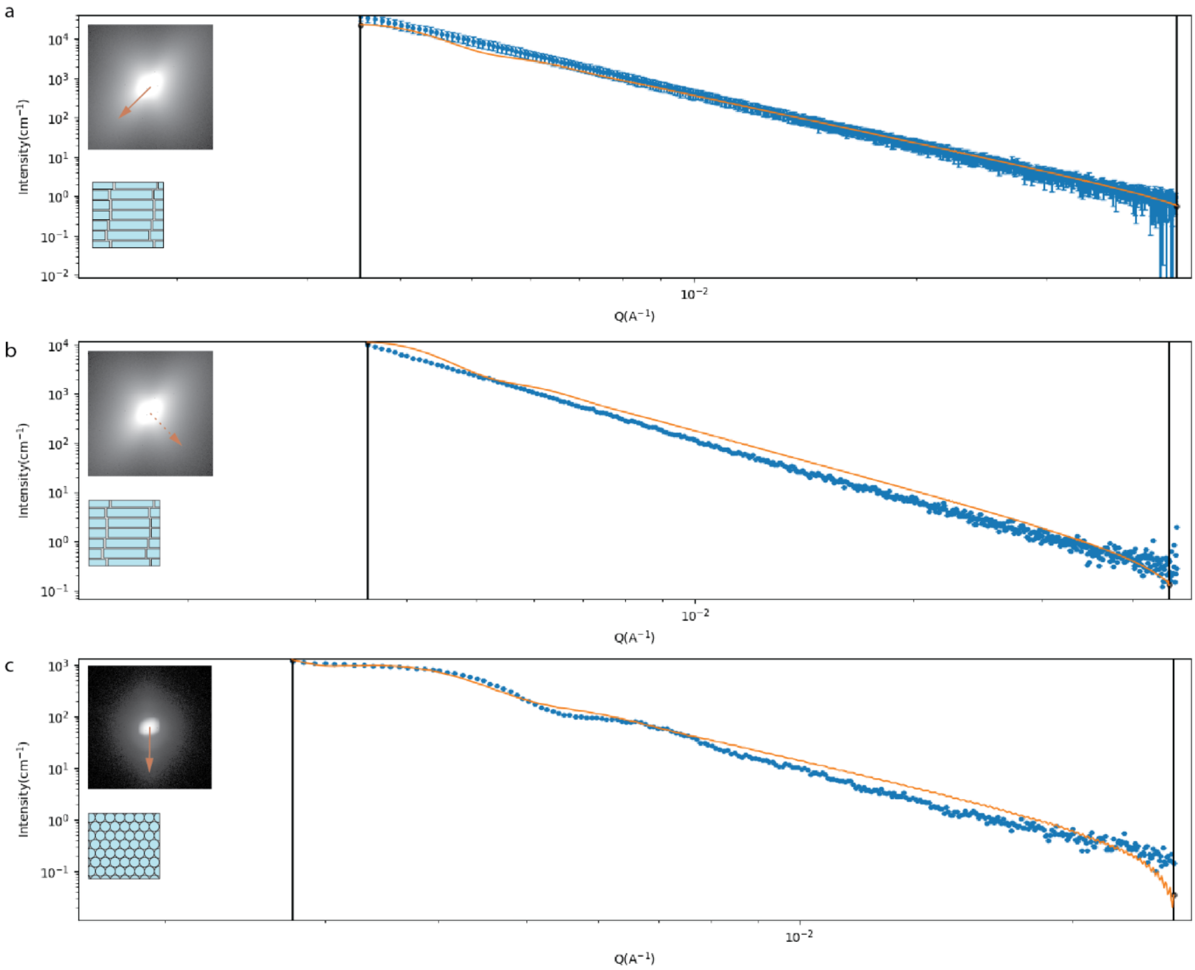

Figure S8. Fitting results using a cylindrical form factor of the SAXS. (a). Intensity as a function of wavenumber in primary direction (see inset). (b). Intensity as a function of wavenumber in the secondary direction (see inset). (c). Intensity as a function of wavenumber with the X-ray going along the direction of the template.

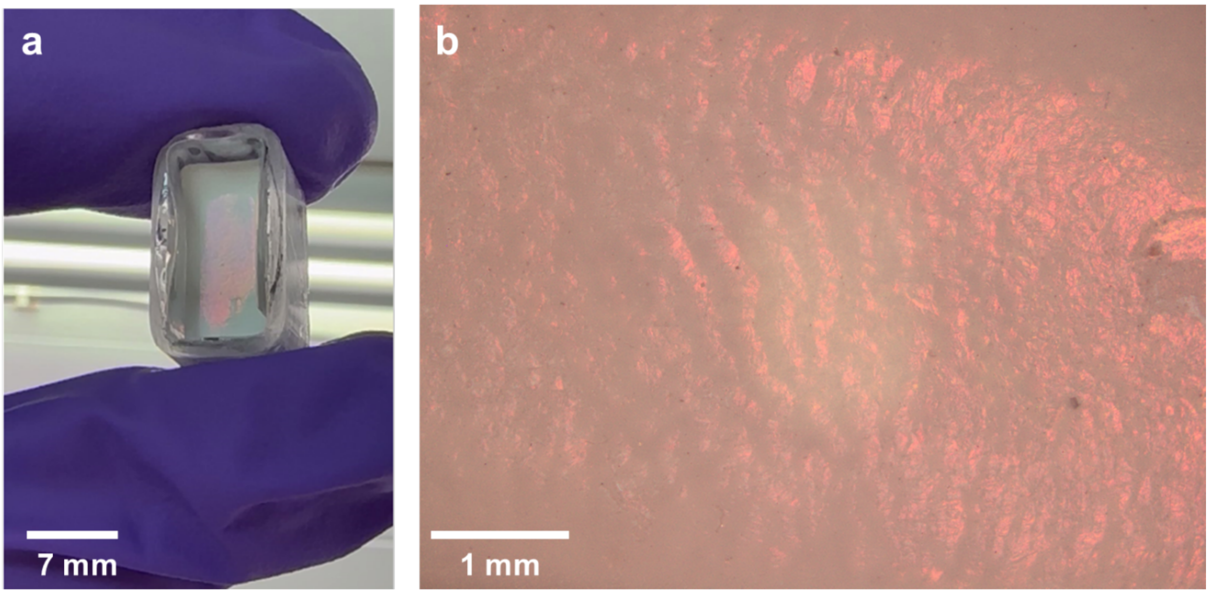

Figure S9. (a) Optical image and (b) microscope image of dried silica rods, showing visible shimmering and structural colours.

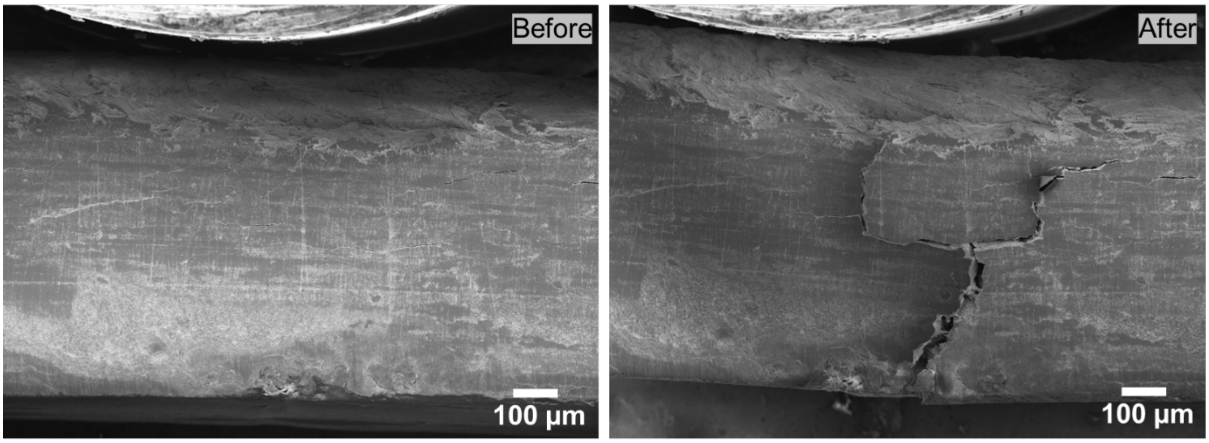

Figure S10. Brittle fracture behaviour of composites with PMMA at the interface.

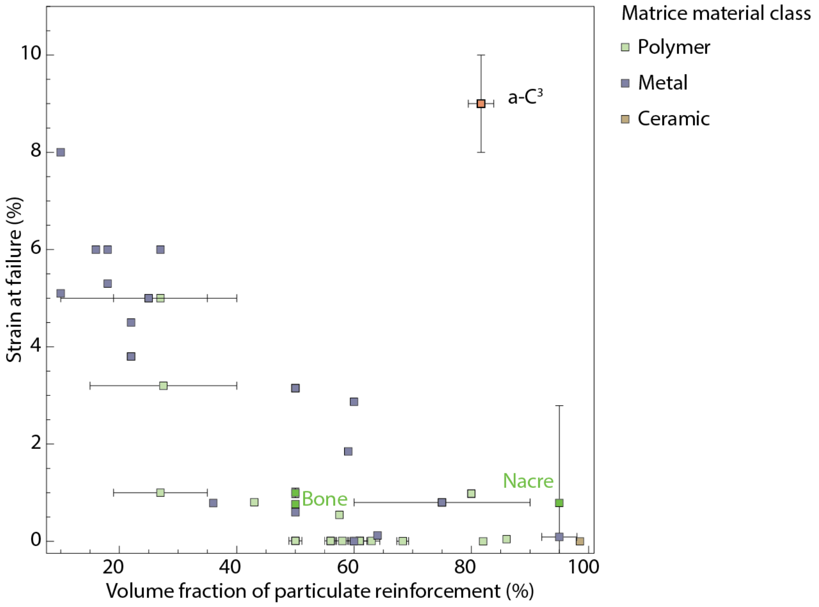

Figure S11. Strain at failure of particulate composites made with different matrices and particulates of different shapes but with sizes below 100μm. Data from references: nacre (5), bone (6, 7), bioinspired composites with polymer (8–13), metal (14–17), ceramic matrices (18). Conventional composites reinforced by particulate SiC or chopped glass fibres and different matrices are taken from the database of the Ansys CES Granta EduPack software.

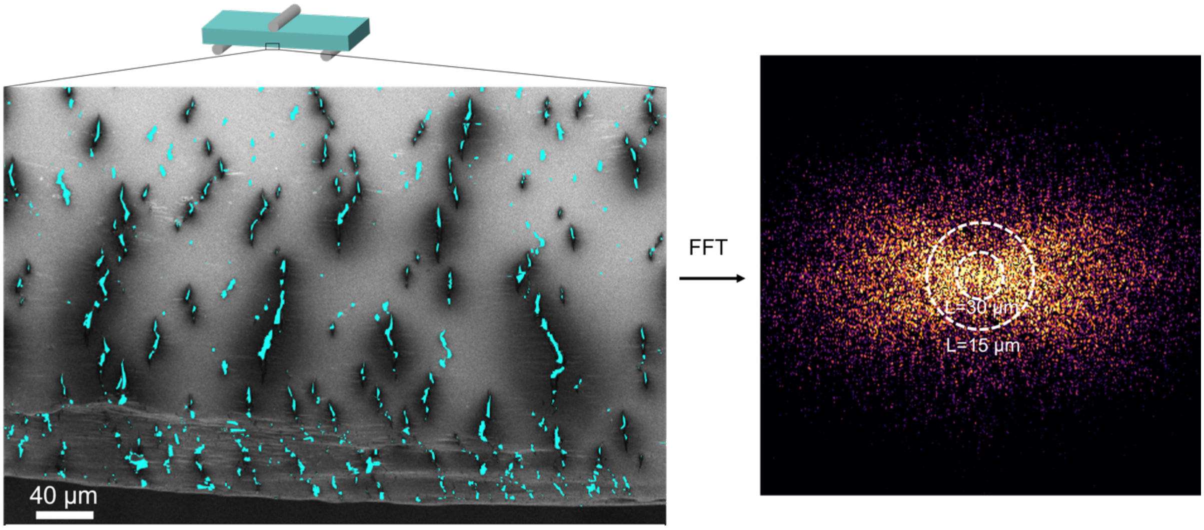

Figure S12: SEM images of the zone in tension taken at ~8% strain during *in situ* bending test of pure PBMA:PMMA polymer. Cracks are highlighted in teal. FFT of the crack network visible in the bottom part of the sample.

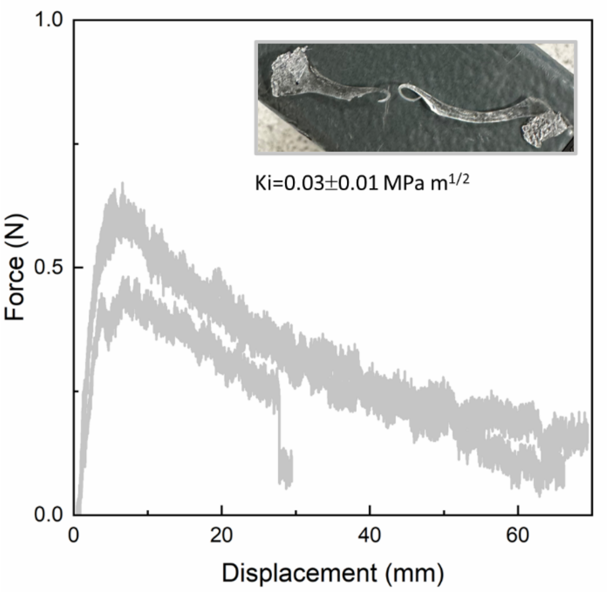

Figure S13. Toughness of the PBMA:PMMA from single edge notch tension test.

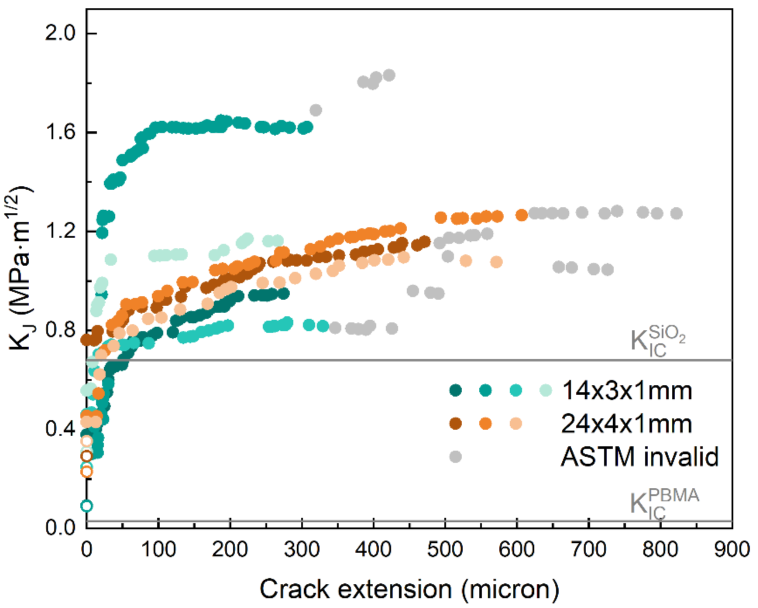

Fig S14. Effect of upscaling sample size on the toughness measured by SENB of $a - C^3|PBMA\!:\!PMMA$.

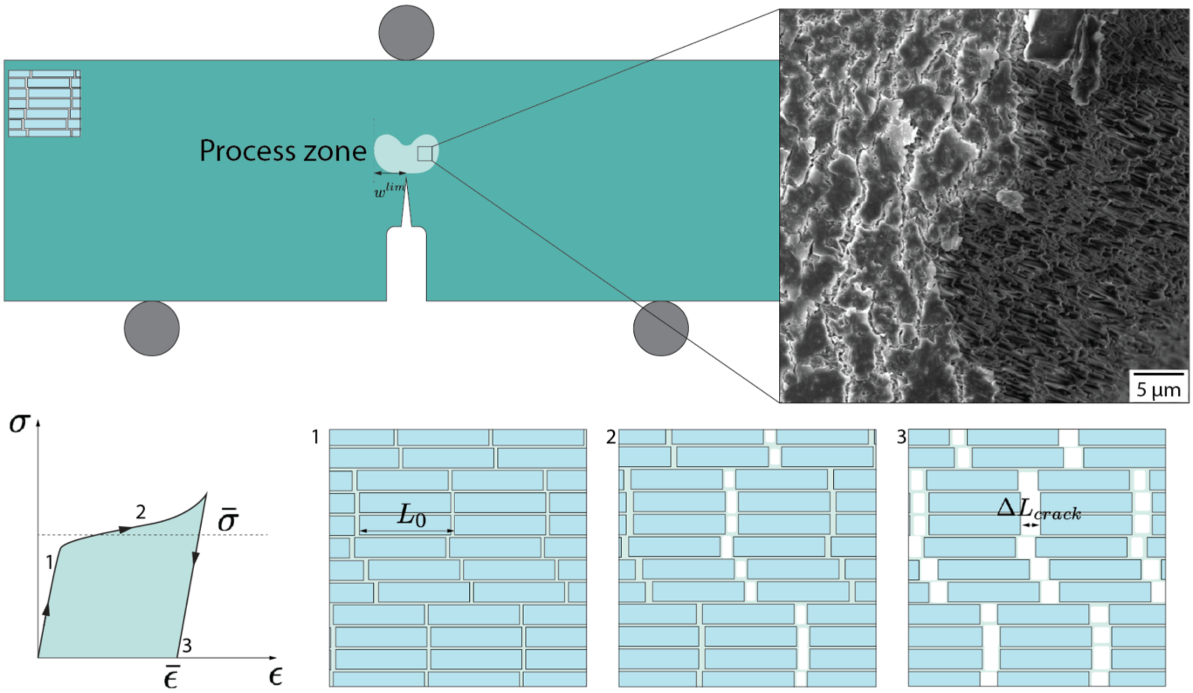

Fig S15. Schematics of process zone toughening of $a - C^3|PBMA{:}\,PMMA$.

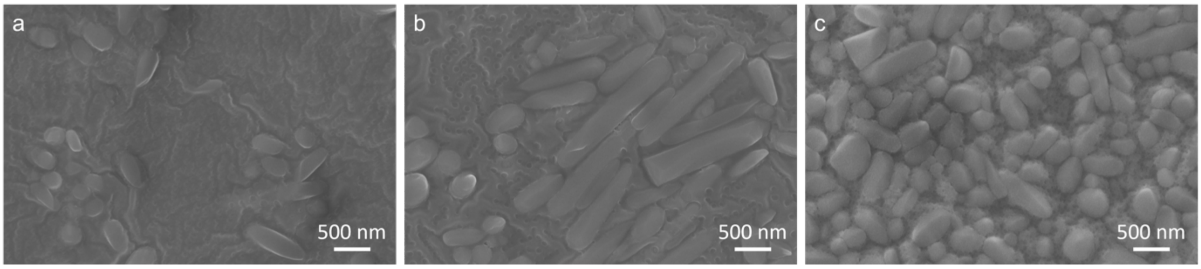

Figure S16: SEM images of composites with (a) 10 vol% and (b) 30 vol% rods by mixing, and (c) 49 vol% rods by centrifugation.

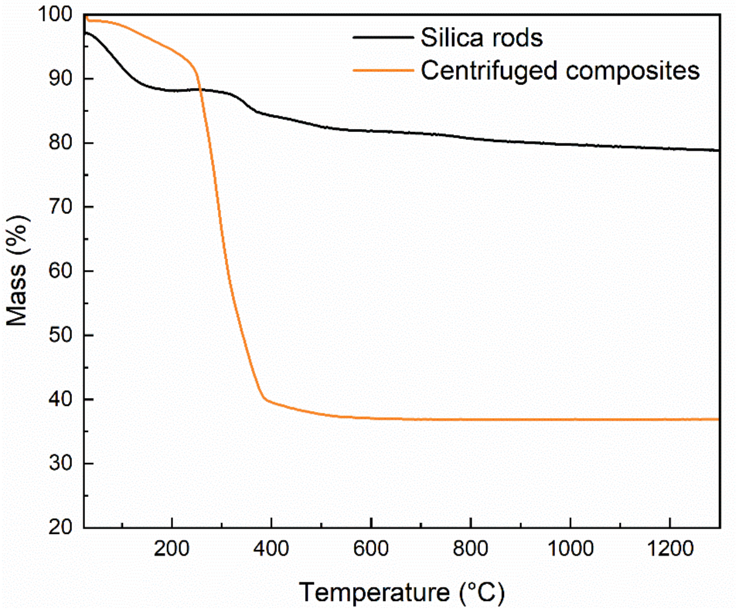

Figure S17. TGA data for silica rods and centrifuged composites.

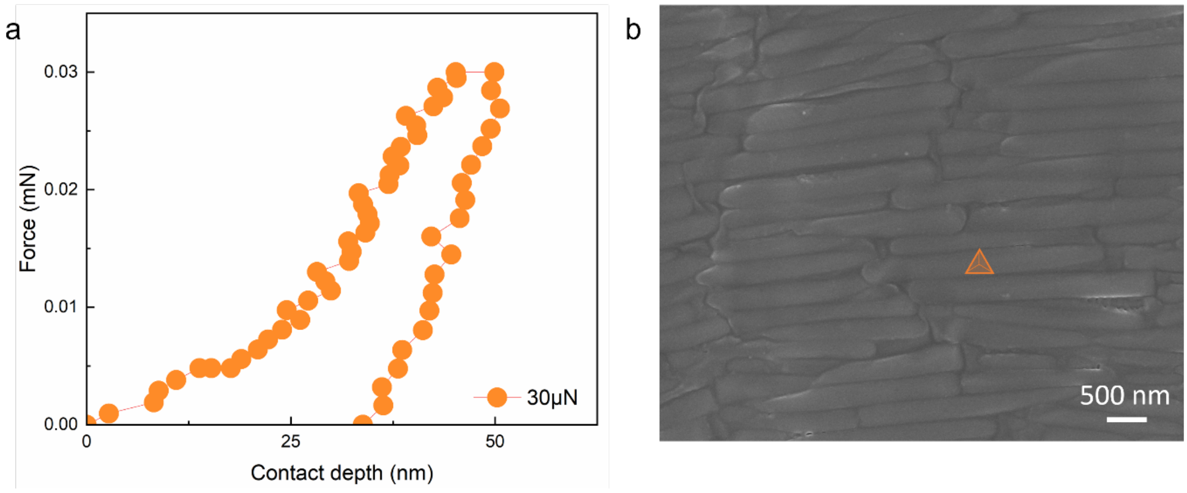

Figure S18. Nanoindentation of a-C³| PBMA:PMMA. (a) Typical indentation profiles at 30 μN showing the maximum indentation depth. (b) Illustration of typical indent sizes at an indentation load of 30 μN.

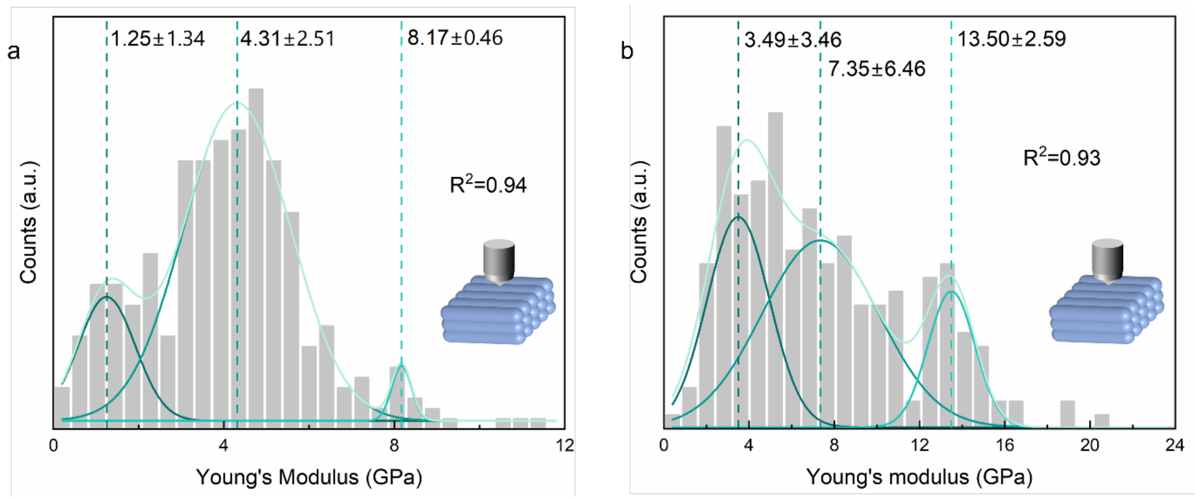

Figure S19. Statistical distribution of Young's modulus measured from 225 indents at 30 μN perpendicular to the rods' length direction. The distribution is fitted by three Gaussian peaks with average and standard deviation given in the plot. (a) a-C^3| PBMA:PMMA with as-synthesised rods, (b) a-C^3| PBMA:PMMA with rods annealed at 600°C.

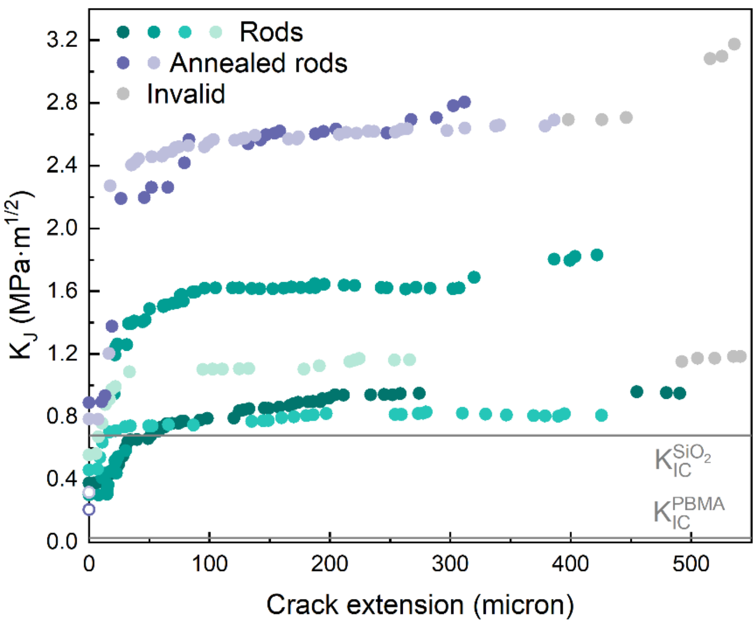

Figure S20. R-curve measured from single edge notch bending tests of a-C³ | PBMA:PMMA made with as-synthesised rods and annealed rods.

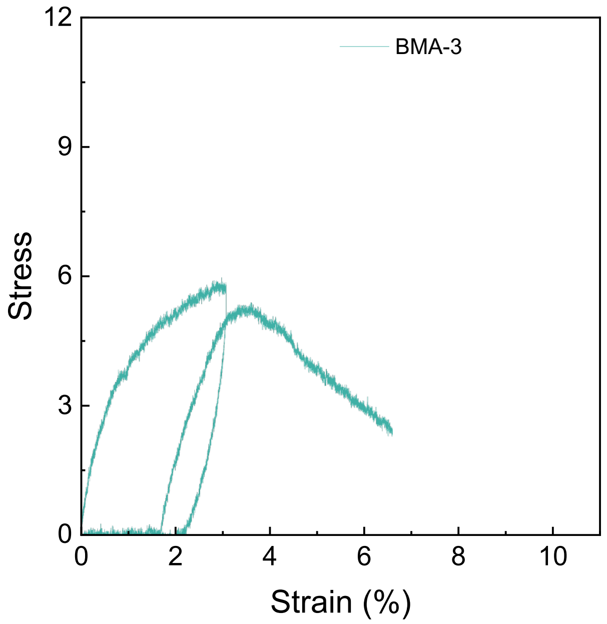

Figure S21. Stress-strain curve of an a-C[3] | PBMA:PMMA in bending with an unloading/loading cycle after 3% strain.

Table S1. Fitting results from SAXS data with a cylinder form factor and polydispersity.

Data profile	Radius (Å)	Polydispersity	Length (Å)	Polydispersity
Primary	1278 ± 51	0.14 ± 0.03	24075 ± 93	0.11 ± 0.01
Secondary	1278 ± 51	0.14 ± 0.03	24075 ± 93	0.11 ± 0.01
Along	1295 ± 5	0.15 ± 0.01	23422 ± 94	0 ± 0.01

References

1. C. B, R. J.R., B. Cotterell, J. R. Rice, Slighlty Curved or Kinked Cracks. *Int J Fract* **16**, 155–169 (1979).
2. F. Barthelat, R. Rabiei, Toughness amplification in natural composites. *J Mech Phys Solids* **59**, 829–840 (2011).
3. B. Lawn, *Fracture of Brittle Solids* (Cambridge University Press, 1993).
4. J. Schindelin, *et al.*, Fiji: an open source platform for biological image analysis. *Nat Methods* **9**, 676–682 (2012).
5. F. Barthelat, H. TANG, P. ZAVATTIERI, C. LI, H. ESPINOSA, On the mechanics of mother-of-pearl: A key feature in the material hierarchical structure. *J Mech Phys Solids* **55**, 306–337 (2007).
6. E. a Zimmermann, B. Gludovatz, E. Schaible, B. Busse, R. O. Ritchie, Fracture resistance of human cortical bone across multiple length-scales at physiological strain rates. *Biomaterials* **35**, 5472–5481 (2014).
7. H. S. Gupta, *et al.*, Cooperative deformation of mineral and collagen in bone at the nanoscale. *Proc Natl Acad Sci U S A* **103**, 17741–6 (2006).
8. S. N. G. Guner, A. F. Dericioglu, Nacre-mimetic epoxy matrix composites reinforced by two-dimensional glass reinforcements. *RSC Adv.* **6**, 33184–33196 (2016).
9. S. N. Gurbuz, A. F. Dericioglu, Effect of reinforcement surface functionalization on the mechanical properties of nacre-like bulk lamellar composites processed by a hybrid conventional method. *Mater Sci Eng C Mater Biol Appl* **33**, 2011–9 (2013).
10. E. Munch, *et al.*, Tough, bio-inspired hybrid materials. *Science (1979)* **322**, 1516–20 (2008).
11. H. Zhao, *et al.*, Multiscale engineered artificial tooth enamel. *Science (1979)* **375**, 551–556 (2022).
12. L.-B. Mao, *et al.*, Synthetic nacre by predesigned matrix-directed mineralization. *Science (1979)* **354**, 107–110 (2016).
13. T. Dörres, *et al.*, Nanoscale-Structured Hybrid Bragg Stacks with Orientation- and Composition-Dependent Mechanical and Thermal Transport Properties: Implications for Nacre Mimetics and Heat Management Applications. *ACS Appl Nano Mater* **5**, 4119–4129 (2022).
14. M. E. Launey, *et al.*, A novel biomimetic approach to the design of high-performance ceramic - Metal composites. *J R Soc Interface* **7**, 741–753 (2010).
15. A. Miserez, A. Mortensen, Fracture of aluminium reinforced with densely packed ceramic particles: Influence of matrix hardening. *Acta Mater* **52**, 5331–5345 (2004).
16. H. Y. Yang, *et al.*, Interface formation and bonding control in high-volume-fraction (TiC+TiB2)/Al composites and their roles in enhancing properties. *Compos B Eng* **209**, 108605 (2021).
17. S. Long, O. Beffort, C. Cayron, C. Bonjour, Microstructure and mechanical properties of a high volume fraction SiC particle reinforced AlCu4MgAg squeeze casting. *Materials Science and Engineering: A* **269**, 175–185 (1999).
18. F. Bouville, *et al.*, Strong, tough and stiff bioinspired ceramics from brittle constituents. *Nat Mater* **13**, 508–14 (2014).
